# Chemical and Technological Aspects of Magnetic Particles for Cancer Liquid Biopsy Applications

**DOI:** 10.1002/open.70249

**Published:** 2026-07-02

**Authors:** Beatriz Naranjo‐Martínez, Ana Laura Coria‐Gutiérrez, Sunčica Sukur, Sedef Ozel‐Okcu, Václav Ranc, Francisco J. Terán, Franca Raucci, Marco A. Pierotti, Domenico Scionti, Anna Piperno

**Affiliations:** ^1^ Cogentech S.r.l. Benefit Company Zona Industriale Catania Italy; ^2^ Department of Chemical, Biological, Pharmaceutical and Environmental Sciences University of Messina Messina Italy; ^3^ Institute of Molecular and Translational Medicine Faculty of Medicine and Dentistry Palacký University and University Hospital Olomouc Olomouc Czech Republic; ^4^ Nanotech Solutions Villacastín Spain; ^5^ Regional Centre of Advanced Technologies and Materials Czech Advanced Technology and Research Institute Palacký University Olomouc Olomouc Czech Republic; ^6^ IFOM ETS ‐ The AIRC Institute of Molecular Oncology Milan Italy

**Keywords:** beads, CTCs, ctNAs, liquid biopsy, magnetic nanoparticles, tumor‐derived EVs

## Abstract

Recent progress in molecular diagnostics has been strongly influenced by advances in magnetic bead (MB) chemistry. Synthetic strategies for MB fabrication play a critical role in defining their size, physicochemical properties, and nano‐ or microscale architectures, which ultimately determine their analytical performance. The efficiency of MBs in liquid biopsy depends on the architecture of their components, including magnetic core, surface functionalization, as well as the integration of recognition ligands. This review highlights the recent developments in MB design for the selective capture, identification, and quantification of cancer biomarkers in liquid biopsy applications. It also summarizes the advantages and limitations of commercially available MB platforms and critically evaluates emerging systems reported in the literature. By comparing current technologies, this review discusses major advances as well as remaining translational bottlenecks, providing guidance for the rational design of next‐generation MBs for liquid biopsy. The latter is an emerging technology increasingly used in precision oncology for molecular profiling to support cancer diagnosis, prognosis, and the selection of personalized treatments.

## Graphical abstract

Magnetic bead engineering for liquid biopsy diagnostics reviews advances in magnetic bead design for the selective enrichment of circulating tumor DNA (ctDNA), circulating tumor cells (CTCs), and extracellular vesicles (EVs) from complex biofluids. The review links surface chemistry, magnetic behavior, and physicochemical properties to analytical performance, highlighting challenges in sensitivity, standardization, and clinical translation toward precision oncology.

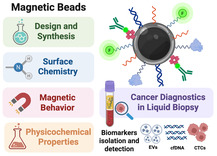

**Abbreviations**
Abs: antibodies
ABEI: N‐(4‐aminobutyl)‐N‐ethyl isopropanol
ALK: anaplastic lymphoma kinase
aM: attomolar
AMF: applied magnetic field
APC: adenomatous polyposis coli
BIBB: 2‐bromoisobutyryl bromide
BMs: biomarkers
bp: base pair
BRCA: breast cancer gene
BSA: bovine serum albumin
cfDNA: cell‐free DNA
cfNA: cell‐free nucleic acids
CLD: cross‐linked Dex shell
CMD: carboxymethyl dextran
CMs: cancer cell membranes
CRISPR: clustered regularly interspaced short palindromic repeats
CSF: cerebrospinal fluid
CTCs: circulating tumor cells
ctDNA: circulating tumor DNA
ctNAs: circulating nucleic acids
Dex: dextran
DNA: deoxyribonucleic acid
EDC: 1‐Ethyl‐3‐(3‐dimethylaminopropyl)carbodiimide
EGFR: epidermal growth factor receptor
ELISA: enzyme‐linked immunosorbent assay
EMT: epithelial‐mesenchymal transition
EpCAM: epithelial cell adhesion molecule
EVs: extracellular vesicles
FDA: Food and Drug Administration
FISH: fluorescence in situ hybridization
fM: femtomolar
FM: ferromagnetic
GMA: glycidyl methacrylate
GSH: glutathione
GST: glutathione‐S‐transferase
H_c_
: coercivity field
HER2: human epidermal growth factor receptor‐2
HETM: hexamethylene tetramine
IMAC: immobilized metal affinity chromatography
IVD: in vitro diagnostics
LMs: leukocyte membranes
MACS: magnetic‐activated cell sorting
MBs: magnetic beads
mRNA: messenger ribonucleic acid
miRNA: micro ribonucleic acid
MNP: magnetic nanoparticle
M_r_
: remanent magnetization
M_s_
: saturation magnetization
mV: millivolt
NCI: national cancer institute
NGS: next‐generation sequencing
NHS: N‐hydroxysuccinimide
nm: nanometer
nM: nanomolar
NPs: nanoparticles
NSCLC: non‐small cell lung cancer
NTA: nitrilotriacetic acid
PAA: polyacrylic acid
PCR: polymerase chain reaction
PEG: polyethylene glycol
pM: picomolar
PM: paramagnetic
PSMA: prostate‐specific membrane antigen
PS‐PMMA: polistyrene‐poli methyl methacrylate
PTMAO: polymeric trimethylamine N‐oxide
RNA: ribonucleic acid
SA: streptavidin
SDS: sodium dodecyl sulfate
Si‐ATRP: surface‐initiated atom transfer radical polymerization
SPCE: single‐particle collision electrochemistry biosensor
SPE: solid phase extraction
SPM: superparamagnetic
SPRI: solid‐phase reversible immobilization
ssDNA: single stranded deoxyribonucleic acid

## 1 Introduction

In the context of precision oncology, liquid biopsies are increasingly used for molecular profiling to support cancer diagnosis, prognosis, personalized treatment selection, and disease monitoring. In parallel, micro‐ and nanotechnology are rapidly advancing, providing novel approaches to enhance the sensitivity and specificity of both diagnostic and therapeutic strategies. Among these developments, the engineering of magnetic beads (MBs) functionalized with recognition ligands for the selective binding of biomarkers (BMs) present in liquid biopsy specimens has emerged as a cornerstone of molecular diagnostics [1].

Cancer represents a leading cause of global mortality, with 20 million new cases and 9.7 million cancer deaths reported in 2022 [2]. The increasing incidence and mortality rates are alarming, with projections suggesting that the number of new cases could reach 35 million annually by 2050, corresponding to a 77% increase compared to 2022 [2]. The global burden of cancer underscores the need for effective strategies to control the disease worldwide, including investments in prevention and early detection to reduce cancer incidence and mortality.

Despite significant advances in cancer therapy that have expanded treatment options, such as targeted treatment and immunotherapy, and improved survival rates, the incidence of several cancers including breast, prostate, pancreas, lung, uterine corpus and colorectal cancers, continues to rise, particularly among younger adults. Many of these malignancies are still diagnosed at an advanced stage, when metastasis and post‐therapy recurrences are more likely to occur, thereby reducing survival and quality of life and incrementing healthcare costs [3]. Addressing these challenges through progress in cancer prevention, early detection, and treatment strategies is essential for improving patient outcomes.

Conventionally, the analysis of genetic alterations has been performed on solid tumor tissues, obtained through a surgical biopsy, for cancer diagnosis and therapeutic decision‐making. However, informative tissue biopsies are invasive, uncomfortable, and not suitable for longitudinal monitoring. In this context, liquid biopsy has emerged as a minimally invasive approach for cancer diagnosis and prognosis prediction through the molecular and cellular analysis of biofluids such as blood, saliva, pleural fluid, ascites, stool, cerebrospinal fluid (CSF), and urine [4, 5]. In parallel, micro‐ and nanotechnology have significantly contributed to advances in cancer diagnostics, particularly through the development of engineered beads designed to selectively bind and capture BMs in liquid biopsy samples.

BM capture using MBs, also referred to as magnetic particles or microspheres, is widely employed for in vitro diagnostic applications, including nucleic acid testing, immunoassays, and cell separation.

**FIGURE 1:**
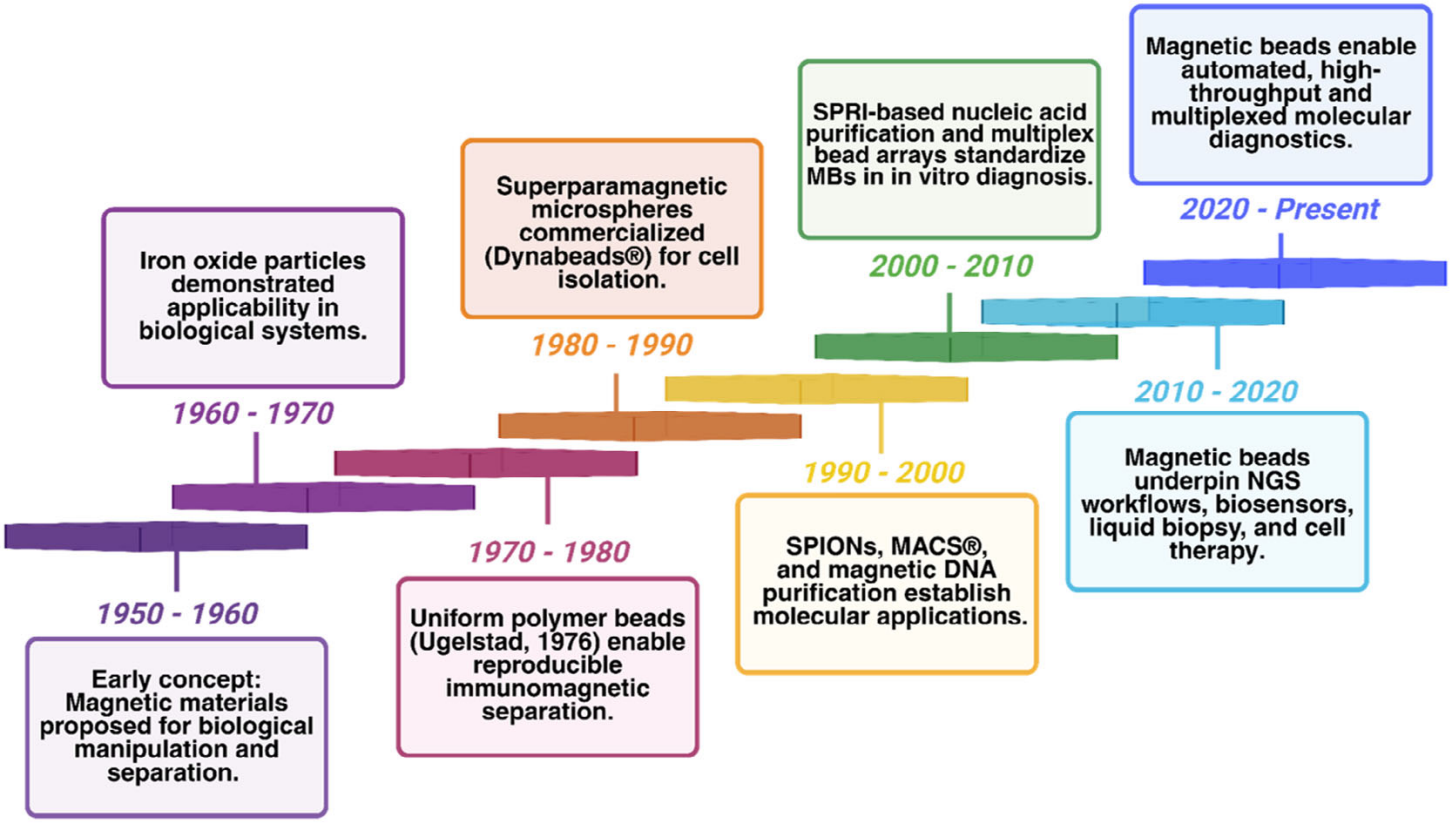
Key milestones of MB advancements in molecular diagnostics. MACS: magnetic‐activated cell sorting; SPRI: solid‐phase reversible immobilization; and NGS: next‐generation sequencing.

Scientific progress in molecular diagnostics is closely linked to advances in MB chemistry (Figure 1). MBs have evolved from conceptual tools to essential components of modern molecular diagnostics. Early studies explored magnetic materials and iron oxide particles for biological manipulation [6], demonstrating that magnetic entities could function in biological systems, including naturally occurring magnetotactic bacteria [7].

The development of uniform polymer microspheres by Ugelstad in the 1970s enabled reproducible immunomagnetic separation and led to the commercialization of superparamagnetic (SPM) beads in the 1980s [8, 9]. During the 1990s, magnetic nanoparticles (MNPs), magnetic‐activated cell sorting (MACS), and the first magnetic DNA purification methods expanded MBs applications into molecular biology [10, 11]. From the 2000s onward, solid‐phase reversible immobilization (SPRI) chemistry and multiplex bead technologies established MBs as standard tools for in vitro diagnostics [12, 13, 14]. More recently, MBs have become foundational for next‐generation sequencing, biosensing, cell therapy manufacturing, and automated high‐throughput and multiplexed molecular diagnostics [15, 16, 17].

**FIGURE 2:**
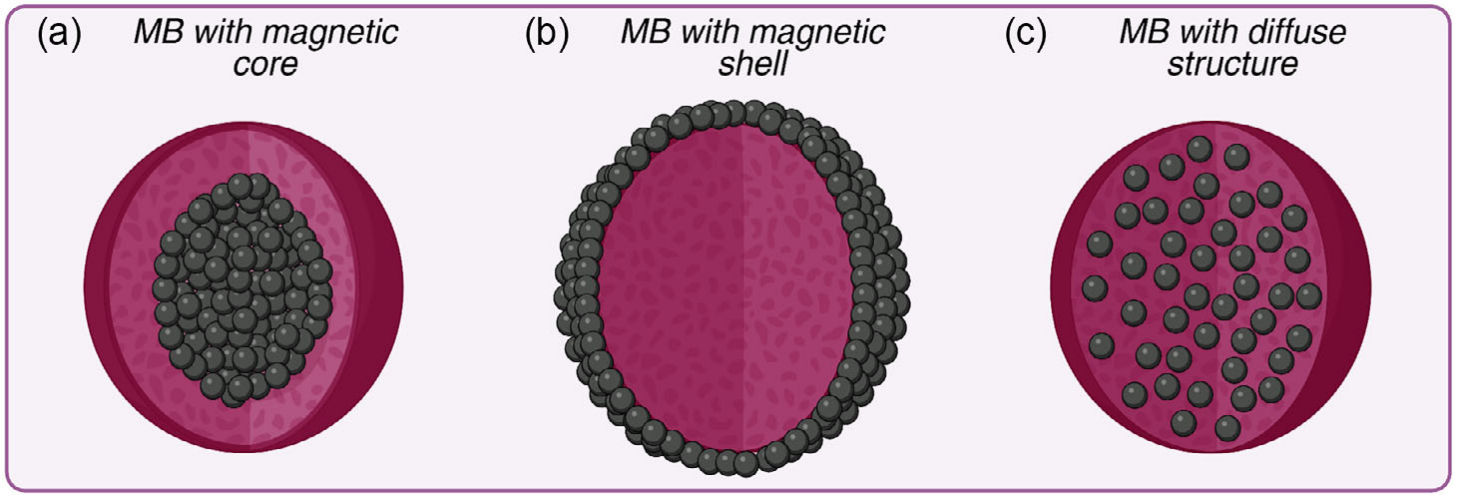
Representation of MBs structure: (a) MBs with magnetic core, (b) MBs with magnetic shell, and (c) MBs with a diffuse structure. Created with BioRender.com.

The effectiveness of MBs relies on the precise design of their key features, including magnetic cores, engineered surface chemistries, and bioconjugation strategies. The size and morphology of MBs are determined by their composition and synthesis methods, leading to particles in the nano‐ or microscale range. The literature describes three main different MB architectures (Figure 2): (a) MBs with a magnetic core, in which magnetite (Fe_3_O_4_) crystals or nanoparticle clusters are coated with organic or inorganic layers; (b) MBs with a magnetic shell, where nonmagnetic particles are coated with MNPs; and (c) MBs with diffuse structure, in which MNPs are uniformly embedded within a matrix, such as agarose [9].

Nanotechnology has increasingly contributed to advances in cancer diagnostics, initially through the development of NPs for the selective capture of BMs and, more recently, through nanotechnology‐based liquid biopsy sensing platforms. These platforms enable minimally invasive sampling and offer powerful tools for cancer diagnosis, disease monitoring, and evaluation of therapeutic responses [18].

The physicochemical configuration of MBs, along with their optical, electronic, and magnetic properties has been widely exploited for the detection of various cancer BMs in many biological fluids [19]. The high surface‐area‐to‐volume ratio, combined with the availability of diverse chemical functionalization strategies, contributes to their high sensitivity, specificity, and stability. Moreover, bead‐based approaches offer higher reproducibility, rapid workflows, easy portability, and low cost compared with other technologies, such as microfluidics or conventional affinity assays [20]. MBs can be functionalized with a variety of biomolecules, including antibodies (Abs) or proteins, to selectively capture and isolate circulating tumor cells (CTCs) [21, 22] or circulating tumor DNA (ctDNA) for early cancer detection [23]. The synthesis, properties, and biological applications of MBs, particularly in nanostructured systems, have been extensively reported in several recent reviews; however, from a chemical perspective, the topic has been addressed only in a fragmented manner. In addition, some aspects of MB chemistry have been discussed within broader reviews focused on the isolation of cancer BMs from biological fluids [24, 25, 26, 27, 28]. This review summarizes the recent advances in MBs for the capture, identification, and quantification of cancer BMs in liquid biopsy and adopts a comprehensive approach to link their chemical, physicochemical, and magnetic properties to biological performance. Design and synthesis criteria, surface chemistry, physicochemical properties, and magnetic behavior are discussed in depth. Finally, the advantages and the limitations of both commercial MBs and emerging MBs reported in the literature are critically analyzed, highlighting translational challenges and their potential to improve cancer diagnostics.

## 2 Liquid Biopsy

According to the definition by the USA National Cancer Institute (NCI), liquid biopsy, from the oncological perspective, involves the analysis of bodily fluid samples to detect cancer‐related DNA, RNA, or other BMs, offering a less invasive alternative to traditional tissue biopsies. Liquid biopsies are useful for screening, early cancer detection, therapy selection, monitoring therapeutic responses, detection of recurrence, and detecting minimal residual disease after treatment [29]. The primary BMs investigated in liquid biopsies include cell‐free DNA (cfDNA) [30, 31], ctDNA [32], cell‐free RNAs (mRNAs, long noncoding RNAs, and miRNAs), CTCs, extracellular vesicles (EVs) [4], tumor‐educated platelets, proteins, and metabolites [33, 34]. These BMs provide information on the genetic and molecular characteristics of tumors, enabling cancer detection and supporting the development of more personalized treatment strategies.

**FIGURE 3:**
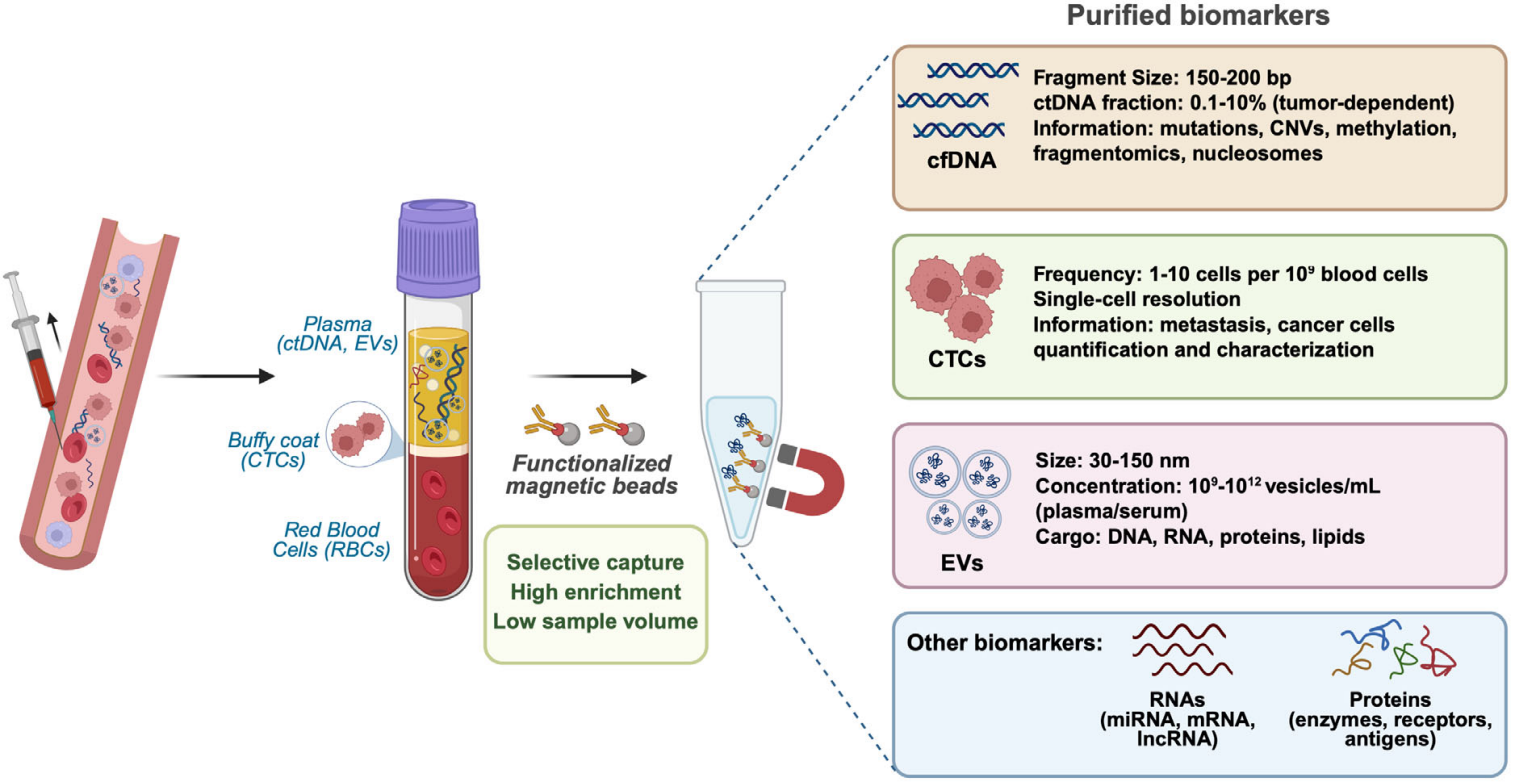
Schematic representation of liquid biopsy workflow using MBs functionalized with recognition ligands. Isolated analytes include cfDNA, CTCs, EVs, as well as RNAs and proteins. Created with Biorender.com.

Additionally, liquid biopsies are crucial for detecting relapse, classifying and localizing tumors, and understanding resistance mechanisms, making them a key tool in personalized medicine [18, 35]. Compared with conventional methods, liquid biopsies offer several key advantages. They require smaller sample volumes, are more cost‐effective, easier to collect, and enable repeated sampling, thereby providing a comprehensive view of disease progression over time. Furthermore, they allow a more comprehensive assessment of the genetic heterogeneity of both primary and metastatic lesions in patients with advanced disease, thus facilitating more personalized therapeutic strategy [1]. In oncology, BMs play a crucial role in identifying cancer type, predicting disease progression, estimating the likelihood of recurrence, and anticipating treatment outcomes. These BMs are valuable for assessing both prognosis independently of treatment (prognostic BMs) and the expected responses to specific therapy (predictive BMs) [36, 37]. ctDNA, CTCs, and EVs represent the most extensively investigated BMs classes in liquid biopsy due to their complementary biological and clinical relevance (Figure 3). ctDNA provides direct access to tumor‐specific genetic and epigenetic alterations and reflects tumor burden, clonal evolution, and molecular heterogeneity. In contrast, CTCs offer information at the single‐cell level by preserving cellular structure and phenotype, thereby enabling functional and molecular analyzes of metastatic disease. EVs, which are abundantly secreted and highly stable in circulation, carry diverse molecular cargo that reflects both tumor‐intrinsic characteristics and interactions with the tumor microenvironment (Figure 3). Moreover, ctDNA has demonstrated significant potential for noninvasive cancer detection, treatment monitoring and prediction of disease recurrence, making it a valuable tool in clinical oncology [38, 39].

## 3 Magnetic Properties of Magnetic Beads

The magnetic response of MBs in biological fluids is determined by both their intrinsic (nanocrystal size, composition, defects) and extrinsic (nanocrystal aggregation, MB concentration) magnetic properties and MB physicochemical conditions. Understanding the interplay between these factors is essential for achieving efficient magnetic manipulation while maintaining colloidal stability in liquid biopsy samples. MNPs may exhibit ferrimagnetic, ferromagnetic (FM) or SPM behavior, depending on the composition and size [40]. The magnetic cores of commercially available MBs are typically composed of magnetite (Fe_3_O_4_), which exhibits ferrimagnetic behavior. A key limitation of ferrimagnetic particles is that they retain magnetization after the applied magnetic field (AMF) is removed, leading to irreversible aggregation and rapid sedimentation when colloidal stability is not optimal in liquid biopsy samples. Paramagnetic particles can overcome this issue, as they do not retain magnetization in the absence of AMF. However, they exhibit lower susceptibility, resulting in weaker magnetic responsiveness, and limiting their utility in separation‐based liquid biopsy applications.

**FIGURE 4:**
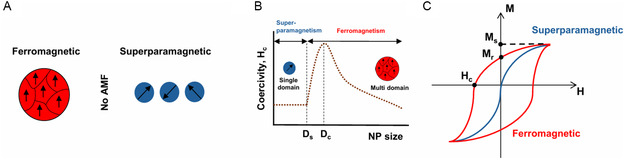
(A) Schematic representation of the ferromagnetic and superparamagnetic behavior for multi‐ and single domain crystals in the absence of an external magnetic field. (B) Relationship between coercivity, particle size and the magnetic domain crystals. *D*_s_ and *D*_c_ are the SPM and critical size thresholds. (C) Typical magnetization curves for FM (red line) and SPM (blue line) NPs. AMF: applied magnetic field; *D*_s_: SPM size; *D*_c_: critical size; *M*: magnetization; *H*: field intensity; *M*_s_: saturation magnetization; *M*_r_: remanent magnetization; *H*_c_: coercivity.

The solution to both aggregation and low magnetic responsiveness lies in reducing the nanocrystal size to achieve SPM behavior [41]. When the magnetic core size is below 15–20 nm, depending on the composition, the nanocrystal becomes smaller than single magnetic domains (Figure 4A,B). In this regime, the magnetic moment of the nanocrystal does not exhibit a preferential orientation, and thermal fluctuations across the magnetic anisotropy barrier drive SPM behavior [42], characterized by zero magnetization in absence of AMF and high magnetic susceptibility under low‐field conditions. The magnetic field strength required to reduce the magnetization value to zero is known as coercivity (*H*_c_). Under an external magnetic field, the nanoparticle magnetic moment rapidly aligns with the field, reaching a saturation magnetization (*M*_s_). As shown in Figure 4C, SPM particles exhibit an anhysteretic magnetization curve M(H), with no coercivity or remanence under static magnetic fields. However, under alternating magnetic fields, hysteretic features emerge due to dynamic magnetization phenomena [42], which underpin many advanced applications of SPM nanocrystals. In contrast, multidomain crystals display open M(H) curves, retaining a magnetic order reflected in the appearance of a remanent magnetization (*M*_r_) in the absence of magnetic fields. These magnetic differences highlight that SPM nanocrystals are essential for the preparation of MBs used in liquid biopsy applications to capture BMs. Furthermore, the combination of high *M*_s_, reduced *H*_c_, and SPM behavior of MBs ensures robust magnetic responsiveness even at low‐intensity fields for the capture of diagnostic targets such as ctDNA, CTCs, and EVs. Table 1 provides a summary of the key magnetic and colloidal properties of the MBs relevant for the liquid biopsy applications.

**TABLE 1:** Colloidal and magnetic properties of various surface‐modified MBs to detect BMs in cancer liquid biopsy.

MBs	Surface	Size (µm)	Ms (emu/g)	BMs	Application	Ref.
**Dyna@MIX MBs**	SA‐Biotinylated DNA	1	23.5	ctDNA	Cancer diagnosis and monitoring	[43]
**HE‐CM‐MNs**	Cell membrane	0.26	63.2	CTCs	BMs isolation from blood samples	[44]
**Fe**_**3**_ **O**_**4**_ **/pGMA/DNA TET/Ti** ^**4+**^	IMAC MBs	0.1–0.2	26.36	EVs	BMs isolation in cancer screening and diagnosis	[45]
**MagStrep Strep‐Tactin MBs**	SA‐tag II‐derived anti‐CD63	1–3	40.5	EVs	On‐chip immunomagnetic BMs separation and purification	[46]
**Fe**_**3**_ **O**_**4**_ **/Au**	Core shell Au	0.3	37	ctDNA	In vitro BMs detection	[47]
**PS‐PMMA/Fe**_**3**_ **O**_**4**_	PS‐PMMA	2	21.51	Protein	Chemiluminescence immunoassay for protein separation and detection	[48]
**Fe**_**3**_ **O**_**4**_ **/Au/Ag/ABEI/Co** ^**2+**^	Core shell Au/Ag/ABEI/ Co^2+^	0.2	55.98	EVs	Label‐free chemiluminescence immunosensor in early cancer diagnosis	[49]
**Lipid MBs**	HER2 ‐ EpCAM	0.2–0.19	18.97	CTCs	BMs capture in disease heterogeneity, clonal evolution, and clonal dynamics	[50]
**FR/Z‐pTA**	Rhm B@ZIF‐8‐pTA	0.313	42.7	CTCs	BMs isolation from blood samples	[51]
**Fe**_**3**_ **O**_**4**_ **/SiO**_**2**_ **/PTMAO**	SiO_2_/PTMAO	0.16	38	CTCs	BMs capture in cancer diagnosis and treatment	[52]
**C6/MMSN‐GPC3**	Glypican‐3	0.11	11.73	CTCs	BMs isolation in early cancer diagnosis	[53]
**AMLV**_**EpCAM**_	EpCAM antibody‐GHDC	0.37	30.97	CTCs	BMs detection in cancer diagnosis and treatment monitoring	[54]
**Fe**_**3**_ **O**_**4**_ **/MgSiO**_**3**_	Magnesium silicate shell	0.25	18	EVs	BMs isolation in early diagnosis and oncotherapy	[55]
**Fe**_**3**_ **O**_**4**_ **/C/anti‐CD63**	Anti‐CD63	0.18	50	EVs	BMs separation and quantification in immunoassays	[56]
**Fe**_**3**_ **O**_**4**_ **/SiO**_**2**_ **/PEI**	Silica/PEI	0.24	29	CTCs	BMs isolation in cancer diagnosis and prognosis	[57]
**CM‐LM‐MBs**	CMs‐LMs	0.1	59	CTCs	BMs detection in a clinical setting	[58]
**Fe**_**3**_ **O**_**4**_ **/PEI**	DNA aptamer	0.35	12.3	CTCs	BMs capture and release for clinical applications	[59]
**Fe**_**3**_ **O**_**4**_ **/SiO**_**2**_	Silica	15–25	2.05	CTCs	BMs detection in cancer treatment	[60]
**AFe**_**3**_ **O**_**4**_ **/SiO**_**2**_	Gold/Silica	0.03–0.05	75	ctDNA	BMs detection in noninvasive early cancer diagnosis and monitoring	[61]
**Fe**_**3**_ **O**_**4**_ **/nSiO**_**2**_ **/mSiO**_**2**_ **/apt**	Silica/anti‐MUC1 aptamer	0.4	59.75	CTCs	Breast cancer cell capture in early cancer diagnosis	[62]

## 4 Chemistry of Magnetic Beads

The magnetic core of MBs, typically composed of magnetite NPs (Fe_3_O_4_ NPs), governs their magnetic behavior. The distinctive magnetite properties, including precise size tunability, biocompatibility, high surface‐to‐volume ratio, and SPM behavior make it highly suitable for bioseparation and diagnostic applications [63]. The magnetic performance of these NPs depends on several structural factors, such as chemical composition, crystal structure, and degree of crystallinity, as well as particle size and shape. Numerous well‐established synthetic strategies for the production of magnetite NPs have been reported in literature, including coprecipitation, microemulsion methods, sonochemical approaches, and hydrothermal synthesis [64, 65]. Each method presents specific advantages and limitations; however, with precise control over reaction parameters, these approaches can yield NPs with the magnetic strength, colloidal stability, and reproducibility required for liquid biopsy workflows.

Uncoated magnetite NPs are prone to surface oxidation and agglomeration; therefore, they are commonly encapsulated within polymeric, inorganic, or other types of shells to produce fully functional MBs. Extensive studies of magnetic nanomaterials have demonstrated that surface engineering is critical for maintaining colloidal stability and preserving magnetic behavior in biological environments [66]. Beyond physical stabilization, surface coatings can also minimize nonspecific protein adsorption, a common source of background signal and false positives, by moderating surface charge, hydrophilicity, and steric repulsion, thereby enhancing assay clarity and capture specificity [66]. Additional MBs for liquid biopsy applications require high stability in complex biological fluids, minimal nonspecific adsorption, and strong chemical and mechanical robustness. Furthermore, MBs must exhibit well‐defined and reproducible surface chemistry to enable reliable biomolecule conjugation under physiological conditions. These factors directly influence separation efficiency, target specificity, and reproducibility in BMs capture workflows. Importantly, these performance parameters are governed by competing factors. For instance, thicker or more complex coatings can enhance stability and increase the density of reactive groups for ligand attachment, but they may also reduce effective magnetization or slow magnetic response due to an increased hydrodynamic size [67]. Consequently, the design of MBs for liquid biopsy requires careful optimization of magnetic performance, surface chemistry, hydrodynamic size, and biological interactions.

**FIGURE 5:**
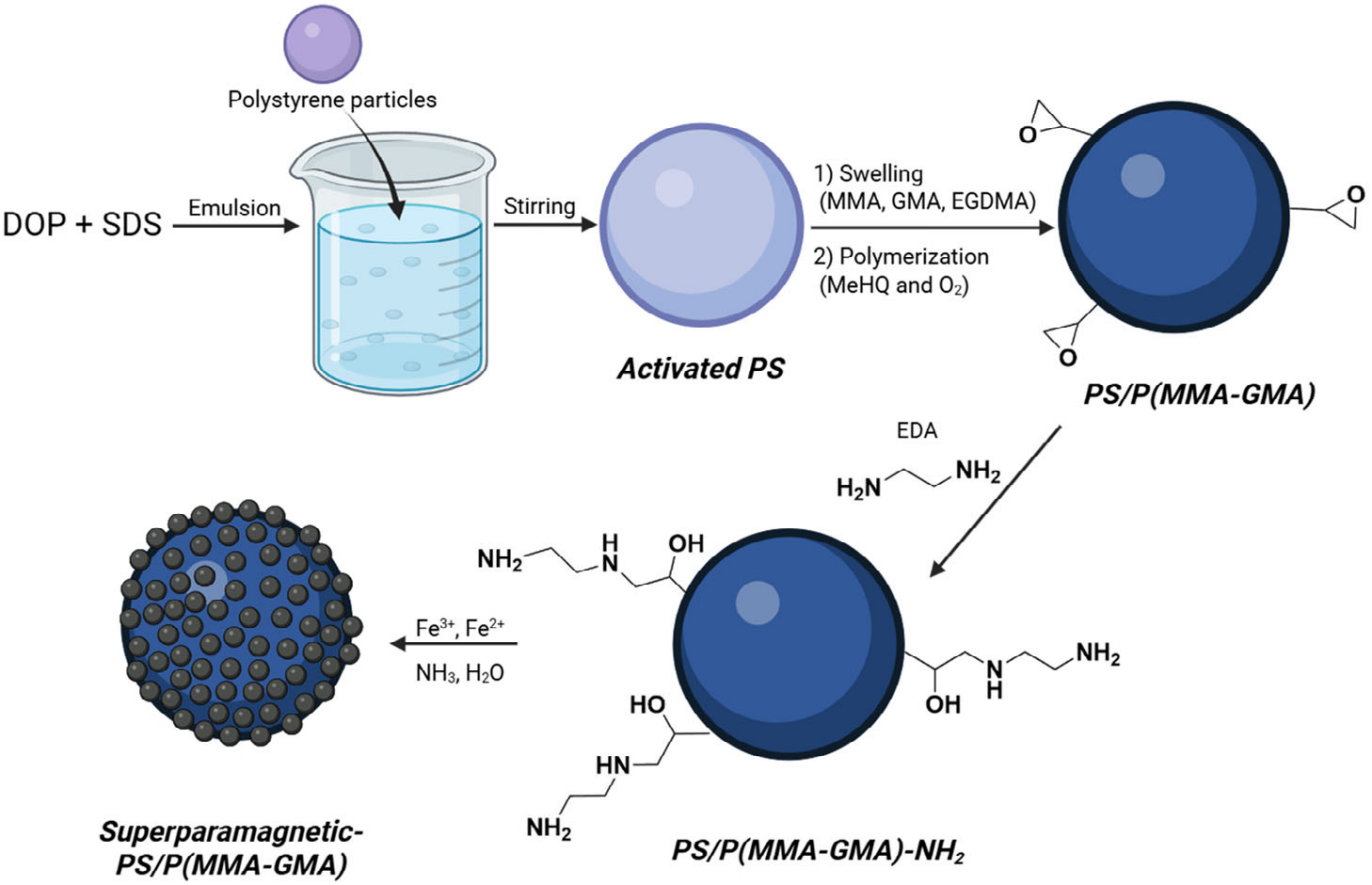
Schematic representation of the synthesis of superparamagnetic‐PS/P(MMA‐GMA) particles through the activated swelling method. SDS: sodium dodecyl sulfate.

Commercial SPM polymer particles with nanostructured magnetite embedded in a polymer matrix are typically produced using the activated swelling method: a modified seeded polymerization technique developed by Ugelstad and collaborators in 1980. This process involves two main steps: (a) activation of seed particles through absorption of a highly hydrophobic compound, and (b) absorption of monomers by the activated seeds followed by polymerization. The resulting particle size and properties depend on the type and quantity of monomers employed. A modification of this strategy, achieved by carefully modulating polymerization kinetics, has been reported by Russo G. et al. to obtain nonspherical, SPM polymer particles. Control over particle shape is attained through the combined use of activated polystyrene seeds, precise adjustment of the swelling ratio, and the simultaneous presence of 4‐methoxyphenol and O_2_ as polymerization inhibitors. This approach enables reproducible transitions from spherical to asymmetric Janus morphologies (Figure 5) [68].

Organic polymers, including polyethylene glycol (PEG), polyacrylic acid, and dextran (Dex) have been investigated to protect the magnetic core, prevent aggregation, minimize nonspecific binding, and provide functional groups for the covalent attachment of recognition ligands, such as Abs, peptides, or oligonucleotides [66].

### 4.1 Silica Coating

Silica has played a defining role in nucleic acid purification for more than four decades. Its ability to bind DNA was first demonstrated in 1979, enabling efficient recovery of DNA from agarose gels [69]. A major milestone was achieved in 1989, when Boom and colleagues introduced a single‐vessel bind‐wash‐elute protocol, providing a rapid and highly reliable means for isolating nucleic acids from complex biological matrices [70]. As the demand for rapid and consistent nucleic acid purification grew in both research and clinical settings, silica became material of choice due to its chemical robustness, low toxicity, affordability, and predictable binding behavior [19]. The transition from bulk silica matrices to silica‐coated magnetic particles was a natural progression, combining silica's favorable surface chemistry with the convenience of magnetic manipulation. This integration enabled rapid DNA/RNA extraction [71], with minimal instrumentation, making it particularly well suited to liquid biopsy settings, where plasma, serum, and urine samples often contain low‐abundance and fragmented nucleic acids.

**FIGURE 6:**
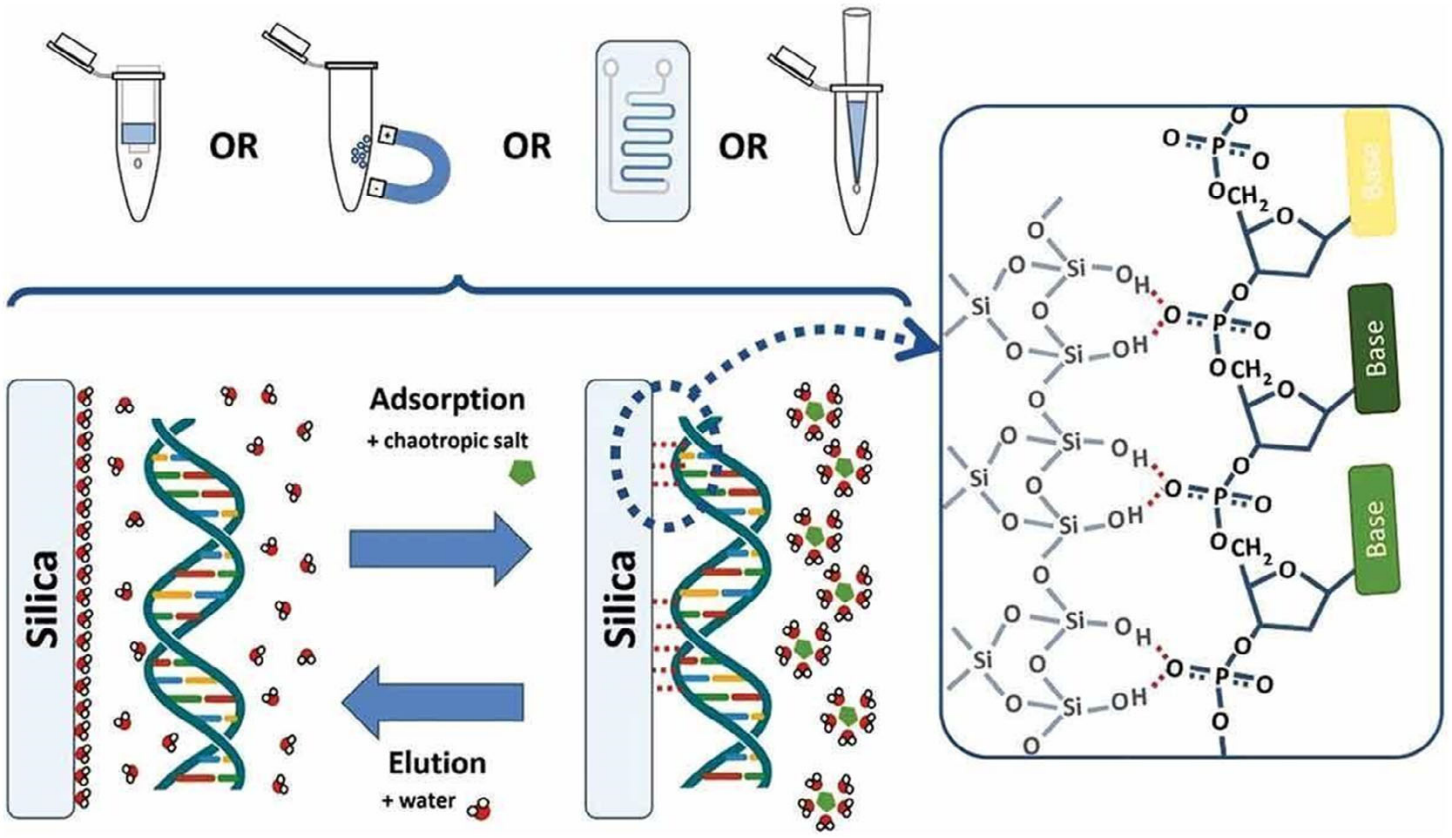
Molecular interactions between nucleic acids and silica surfaces. Reproduced with permission [72]. Copyright 2023, Taylor & Francis.

Silica binds nucleic acids through interactions between surface silanol groups and the phosphate backbone of DNA (Figure 6). Computational studies by Shi and Shin indicate that adsorption is driven by a cooperative network of hydrogen bonds supported by hydrophobic interactions [73]. The presence of chaotropic salts further enhances this process by disrupting the hydration shell around DNA and promoting its dehydration, making adsorption thermodynamically favorable. Elution is typically achieved under low‐salt conditions, above the isoelectric point of silica, where electrostatic repulsion promotes release.

A range of synthesis strategies have been developed to deposit silica onto magnetic cores. The Stöber sol–gel method is scalable and produces uniform shells; microemulsion approaches allow precise control over shell thickness but are more complex and less scalable; and aerosol pyrolysis is suitable for large‐scale production albeit with less control over shell uniformity [74]. In addition to providing a binding interface for nucleic acids, silica coatings protect MNPs from oxidation, improve aqueous dispersibility, enhance biocompatibility, and create a chemically versatile surface for subsequent functionalization [61].

**FIGURE 7:**
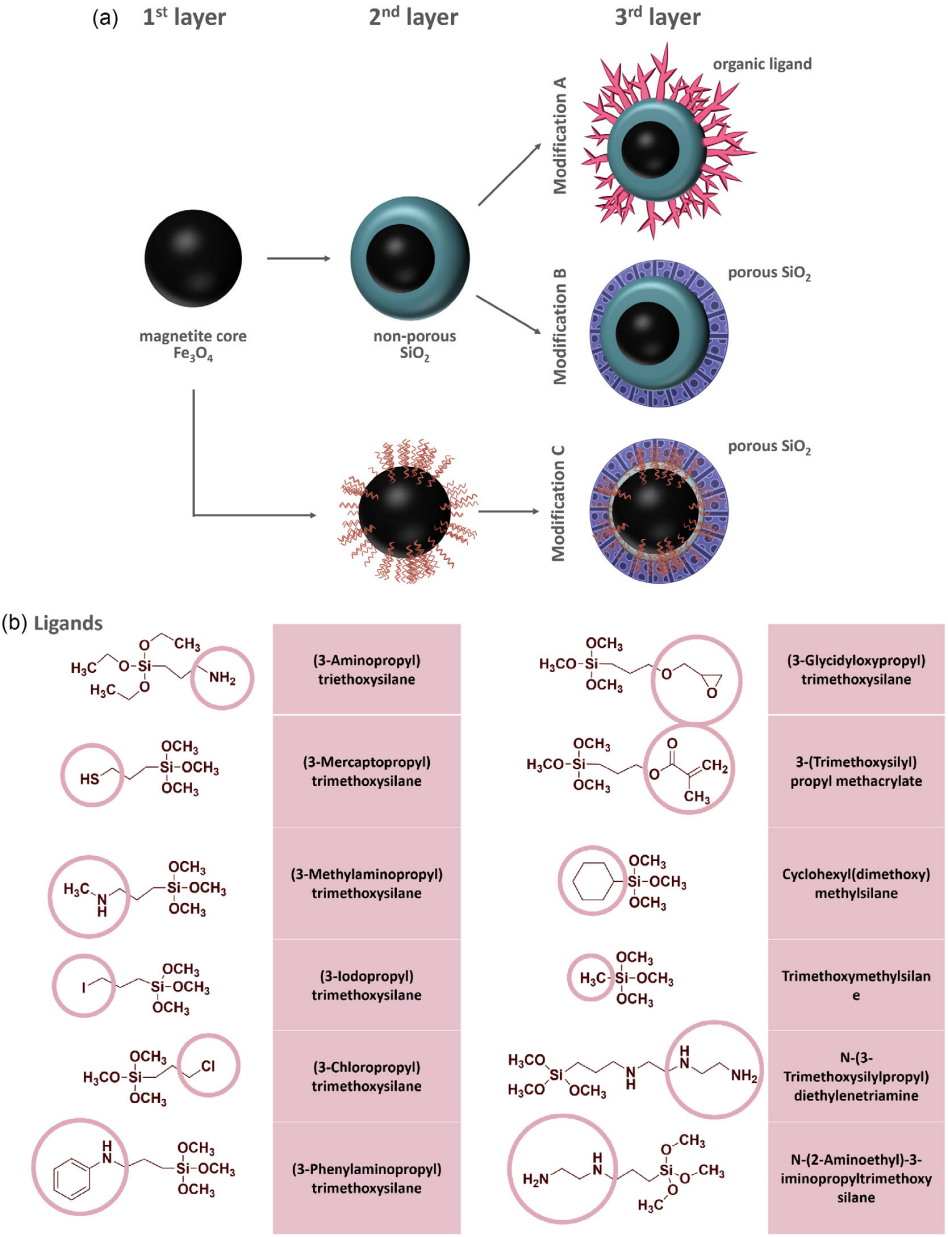
Three‐step strategy for the preparation of silica MBs. (a) Surface functionalization via organic ligands (Strategy A), deposition of a porous silica layer on silica‐coated magnetite NPs (Strategy B), or porous silica growth on oleic‐acid‐coated magnetite NPs (Strategy C). (b) Examples of organic ligands investigated for surface modification of MBs. Adapted with permission [67]. Copyright 2024, Sci Rep.

Recently, Zeleňáková et al. reported a comprehensive study on the preparation of different types of silica‐coated MBs for the isolation of viral RNA [67]. Their three‐step synthetic strategy included: i) the synthesis of magnetic core; ii) coating with a nonporous layer of SiO_2_ or oleic acid; and iii) surface functionalization using organic ligands or a porous SiO_2_ layer (Figure 7).

Silica has been employed to synthesize size‐controlled mesoporous silica MNPs (MSCMNPs) for extracting extracellular DNA. The mesoporous structure of MSCMNPs, with a pore size of ~3.0 nm, allows the effective capture of even highly fragmented DNA (less than 100 bp) without the need for chaotropic agents. This high DNA affinity ensures excellent extraction performance, with capacities reaching 833.3 mg/g, while also resisting interference from proteins, humic acids, and other compounds present in the samples [75].

Despite these advantages, silica exhibits inherent limitations. A small fraction of nucleic acid often remains irreversibly bound to the silica surface after elution, which prevents bead reuse and reduces the effective binding capacity [72]. Moreover, extraction efficiency strongly depends on buffer composition, chaotropic salts and pH, as silica alone provides inherent target selectivity without additional functionalization. Nevertheless, these limitations are outweighed by silica's numerous strengths. It is chemically stable, mechanically robust, inexpensive, and easily modified via silane chemistry, consistently delivering reproducible nucleic acid recovery across a range of commercially available materials (Table 2). Silica‐coated MBs are particularly effective for low‐abundance and fragmented nucleic acids, such as cfDNA and ctDNA fragments [72].

**TABLE 2:** Highlighted commercially available silica‐coated MBs with main properties.

MB surface	**Size** (µm)	Ms (emu/g)	Properties	Application	Ref.
Hydroxyl (—OH)	0.25–1.5	SPM	Hydrophilic, chemically stable, easy magnet separation. Suitable for silanization or nucleic‐acid binding	DNA/RNA adsorption, SPE, chemical surface modification	[76, 77, 78, 79]
	0.150–1.5 (Polydisperse)	FM	SiO_2_/Fe_3_O_4_; solids 2.5%; dispersed in water; polydisperse	Viral nucleic acid extraction	
Amine (—NH_2_)	0.25–1.5	>63	∼150–300 nmol COOH‐reactive sites/mg beads (EDC/NHS coupling)	Protein and Abs conjugation	
Carboxyl (—COOH)	0.25–1.5		∼200–400 nmol NH_2_/mg beads (EDC/NHS coupling)	Protein and Abs conjugation, flow cytometry, nucleic acid purification	
Epoxy	1.5	>8	Moderate protein coupling (∼5–15 mg protein/mL beads)	Protein and Abs conjugation	[76, 77]
NHS‐activated	1.5		Activated ester chemistry	Protein and Abs conjugation	
NTA/Ni‐NTA	1.5		Metal chelation for His‐tag binding, ∼20–40 mg His‐tag protein/mL beads	Affinity purification of His‐tagged proteins	
Avidin/Streptavidin/Biotin	1.5	SPM	High affinity biotin binding, ∼300−500 pmol biotin/mg beads	Immobilization of biotinylated molecules	[76]
Protein A/Albumin	1.5	SPM	Specific protein binding/blocking, IgG Fc binding (Protein A); BSA for blocking	Abs purification	
Octadecyl (C18)	0.25–1.5	>8	Lipophilic interactions, hydrophobic binding	Nucleic acid purification, SPE, reverse‐phase chromatography	
Special chemistries (Phenyl + Pyrrolidine Ketone, TiO_2_, UFH)	3–5 µm (Phenyl + Pyrrolidine Ketone)	SPM	Lipophilic, phosphopeptide affinity, nucleic acid binding	SPE, phosphopeptide enrichment, anticoagulant protein binding	
Plain/not functionalized	0.25–1.5	>8 (75%–80% magnetite)	Hydrophilic, chemically stable, base OH surface, low binding	Nucleic acid adsorption, SPE, general laboratory use	[77, 78]
Silica (silanol)	2.5–4.5	Fe_3_O_4_ core	50 mg/mL; density ∼1.03 g/cm^3^, >4 mg DNA/mL beads	Plasmid DNA, genomic DNA, RNA for qPCR	[80]

Beyond conventional purification, silica‐magnetic composites have become increasingly important in biosensing technologies. Their high surface area, stability, and compatibility with functional nanomaterials have enabled the development of sensitive detection platforms capable of identifying ctDNA, miRNA, and EVs at extremely low concentrations [81]. Silica‐coated MBs have also shown potential in clinical applications, such as noninvasive prenatal testing, where they support reliable isolation of cfDNA from maternal plasma [82]. Taken together, silica coatings provide a robust and versatile interface between magnetic separation and nucleic acid chemistry. Their stability, compatibility with complex biological matrices, and proven efficacy in both extraction and biosensing underscore their prominence in liquid biopsy applications, including those requiring the sensitive detection and characterization of methylated DNA.

### 4.2 Dextran Coating

**FIGURE 8:**
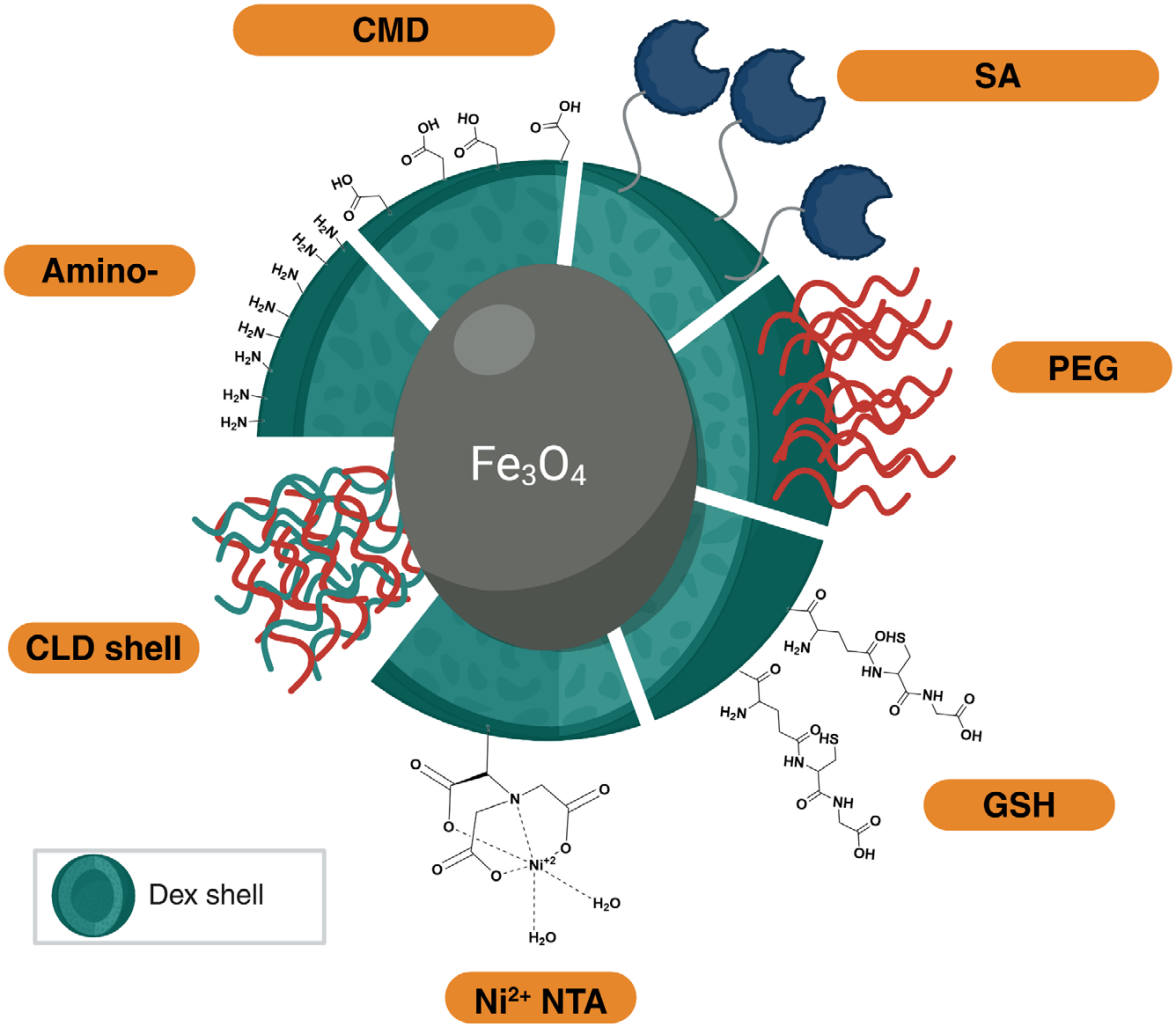
Dex MBs with different functionalization. Created with Biorender.com.

Dex is a hydrophilic glucose‐based polysaccharide mainly composed of α‐1,6‐linked glucose units with occasional α‐1,3 branches. It is typically adsorbed onto MNP surfaces via noncovalent interactions under alkaline conditions [83]. The biocompatibility, nonimmunogenicity, and hydrophilic nature of Dex make it one of the most widely used coatings for biomedical MNPs. Dex shell enhances colloidal stability, minimizes nonspecific protein adsorption, mitigates aggregation, and reduces cytotoxicity compared to bare iron oxide. Furthermore, oxidation or chemical modification of Dex introduces reactive functional groups (i.e., OH, COOH, NH_2_, and CHO), enabling subsequent conjugation with biomolecules such as Abs, aptamers, peptides, or oligonucleotides [84]. The physicochemical versatility of Dex allows the generation of multiple functional derivatives, each imparting surface chemistry and binding properties (Figure 8).

**FIGURE 9:**
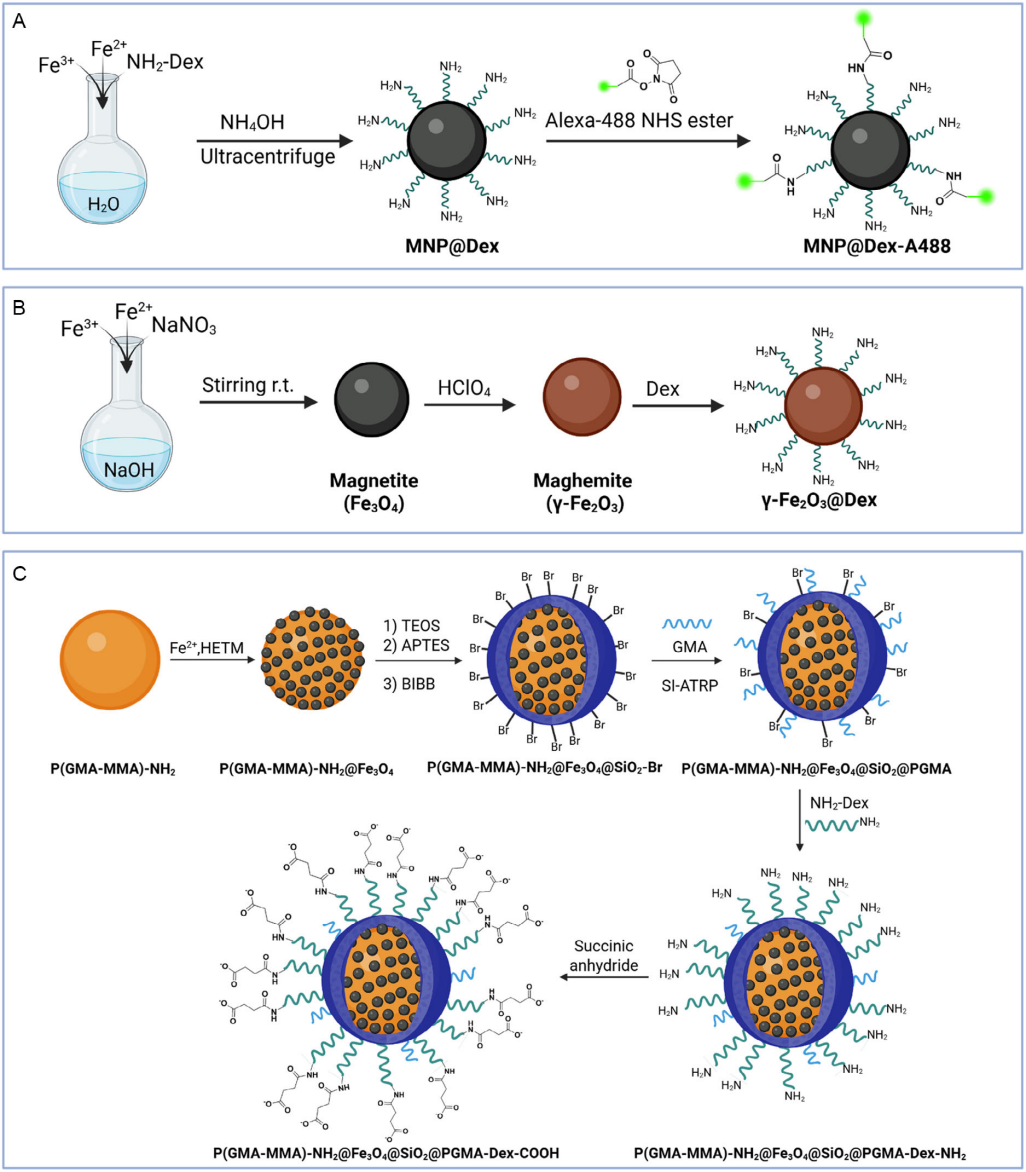
Synthesis of Dex MBs. (A) MNP@Dex [85]. (B) γ‐Fe_3_O_2_@Dex [86]. (C) P(GMA‐MMA)NH_2_@Fe_3_O_4_@SiO_2_@PGMA‐Dex‐NH_2_ [87]. BIBB, 2‐bromoisobutyryl bromide; GMA, glycidyl methacrylate; HETM, hexamethylene tetramine; and Si‐ATRP, surface‐initiated atom transfer radical polymerization. Created with Biorender.com.

The main synthetic routes for the preparation of Dex‐coated MNPs include: i) in situ coprecipitation of iron salts in the presence of Dex; ii) thermal decomposition of organometallic precursors followed by ligand exchange and Dex grafting; iii) postsynthesis coating of preformed MNPs via adsorption or covalent attachment of Dex or carboxymethyl dextran (CMD) [85, 86]. Naha et al. synthesized Dex‐coated MNPs by coprecipitation of Fe^2+^/Fe^3+^ ions in the presence of polymers, followed by thermal treatment and purification to obtain stable, functional NPs (Figure 9A) [85]. Predoi et al. developed Dex‐coated iron oxide magnetic fluids by three‐step procedure: i) coprecipitation of iron salts, ii) oxidation of magnetite to maghemite; iii) Dex‐coating to provide steric stabilization and aqueous dispersibility (Figure 9B) [86]. Su et al. fabricated Dex‐grafted magnetic polymer microspheres using poly(glycidylmethacrylate‐methylmethacrylate) (P(GMA‐MMA)) scaffolds, followed by MNP incorporation, silica coating, and surface‐initiated atom transfer radical polymerization (ATRP) to grow dense poly(glycidylmethacrylate (PGMA) brushes. Subsequently, Dex grafting and carboxylation yielded highly dispersible, magnetically separable microspheres with low nonspecific protein adsorption, suitable for selective bioseparation and immunoassay applications (Figure 9C) [87].

The size of MNPs produced using the mentioned synthetic strategies ranges from ~10 nm to several hundred nanometers (hydrodynamic diameter), depending on synthesis conditions and intended application. However, these systems still face challenges, including batch‐to‐batch variability, nonspecific adsorption, and difficulties related to large‐scale production and sterilization.

Historically, Dex‐coated MBs such as Feridex (ferumoxides) were among the first iron oxide agents approved for MRI contrast enhancement [88]. More recently, modified Dex‐coated MBs have been increasingly adapted for liquid biopsy applications, where they serve as robust solid support for the capture of circulating BMs (DNA, proteins, EVs, and cells). Today, a wide portfolio of Dex‐coated MBs is commercially available, representing a well‐established class of MBs for bioseparation and diagnostic applications (Table 3).

**TABLE 3:** Representative types of commercially available Dex‐coated magnetic particles. The information displayed in this table is collected from the suppliers’ websites. NTA, nitrilotriacetic acid.

MBs	Surface	Size (nm)	Ms (emu/g)	Properties	Application	Ref.
CMD	COOH groups, Dex shell	15–500	From 51 to 70–75	Covalent coupling of amine‐ligands; stable in aqueous media. Covalently binding of Abs, aptamers, peptides.	Capture of CTCs or tumor‐derived EVs. Magnetic isolation of cfDNA and miRNA. Immunomagnetic enrichment of specific BMs.	[89]
Amino Dex MBs	NH_2_	100–500	>70–110	Grafting of carboxyl‐bearing ligands; high surface reactivity, Suitable for Abs and proteins conjugation.	Electrostatic capture of cfDNA/RNA; EVs/CTC separation by grafted Abs or ligands. Multiplex immunomagnetic separation in microfluidic devices.	[90, 91, 92]
CLD (cross‐linked Dex shell)	–NH_2_, –COOH, PEG‐COOH options	300–500	>70	High mechanical stability; low ligand leaching; uniform shell.	Reusable support during EV and cfDNA extraction. High‐throughput plasma processing for bulk isolation of EVs and exosomes.	[93]
Nickel(II)–NTA Dex MBs	Dex shell with Ni^2+^–NTA chelator groups	100–500	>70–75	Selective capture of His‐tagged proteins or ligands.	BMs enrichment or protein‐DNA interaction assays.	[94, 95]
PEG Dex	Dex and PEG linkers with different end groups	50–500	>63–75	Improved ligand access; reduced steric hindrance; lower nonspecific adsorption; improved colloidal stability in plasma.	EVs and cfDNA isolation; magnetic enrichment of low‐abundance BMs; Integration on microfluidic chips for EVs and nucleic acids separation.	[96, 97]
GSH Dex	GSH	130–250	>63–75	High affinity to glutathione‐S‐transferase (GST).	Purification of GST‐linked probes or biosensors used in cfDNA detection assays.	[98, 99]
SA Dex	SA	100–500	>75	High affinity to biotinylated probes	Biotin‐based capture of cfDNA, EVs, or CTCs; digital ELISA and cfDNA quantification.	[100]

CMD particles provide reactive carboxyl groups for covalent ligand coupling, supporting selective capture of CTCs, EVs, and cfDNA binding probes. Amino Dex MBs allow straightforward conjugation with carboxylated ligands and facilitate electrostatic adsorption of nucleic acids; however, their positive charge may increase nonspecific interactions. CLD coatings enhance structural robustness and minimize ligand leaching, enabling reliable performance under high‐throughput or automated separation conditions. Nickel(II)‐NTA‐Dex MBs allow affinity capture of His‐tagged biomolecules, while PEG‐Dex composites provide antifouling and hydrophilic properties that enhance selectivity in complex biofluids. Glutathione (GSH) or streptavidin (SA) Dex MBs are widely used for magnetic capture of glutathione‐S‐transferase (GST)‐ or biotin‐tagged cfDNA probes, EVs, and proteins.

Although Dex‐derivative MBs are commercially available, new synthetic and formulation strategies continue to be developed. Xu et al. synthesized CMB Dex MBs by solvothermal method, followed by covalent functionalization with SA. After the interaction with biotinylated anti‐human CD3 antibody, these MBs were used to isolate CD3^+^T cells, achieving a cell purity of 85.2% and a recovery efficiency of 61.5% [101]. Collectively, the literature indicates that Dex‐coated MBs combine chemical tunability and magnetic responsiveness, providing modular and reproducible tools for the selective enrichment and analysis of circulating BMs in liquid biopsy assays.

### 4.3 Polystyrene Coating

Polystyrene is one of the most established polymeric materials used in bioanalytical technologies due to its chemical stability, hydrophobicity, and compatibility with a wide range of surface‐modification strategies. Although inherently nonporous and chemically inert, polystyrene can be functionalized with carboxyl, amino, epoxy, chloromethyl, or GMA groups, enabling reliable biomolecule immobilization as well as passive adsorption‐based interactions [102]. These properties emphasize its longstanding use in microbeads, microwell plates, biosensor platforms, and MBs. Although many commercially available MBs adopt a polystyrene core with a magnetic shell structure, a substantial body of work also describes the inverse structure configuration [103]. Table 4 presents some representative examples; these structures are particularly important in liquid biopsy because the polystyrene shell provides a chemically versatile, robust, tunable, and easily functionalized interface for the grafting of recognition ligands. Given their importance in the selective BM capture, this class of materials is in‐deep discussed in this review. Various polymerization routes have been explored for the preparation of MBs with polystyrene shells. One strategy involves the entrapment of magnetite MNP within a low‐crosslinked polystyrene matrix, resulting in beads that preserve polystyrene's characteristic swelling behavior while remaining magnetically separable [108]. More commonly, the loading of Fe_3_O_4_ NPs occurred during emulsion, suspension, or dispersion polymerization. In classical seeded or emulsion polymerization, Fe_3_O_4_ NPs are embedded during styrene polymerization, optionally together with comonomers such as GMA, which introduces reactive epoxy groups for downstream probe attachment. This approach allows the formation of stable, functionalized microspheres such as the polystyrene/GMA beads described by Chung et al., which were further modified with DNA probes for hybridization‐based capture [109]. Microsuspension polymerization similarly yields spherical 1–10 μm beads with good sphericity, strong magnetic responsiveness, and broad solvent compatibility, as demonstrated in the synthesis of Fe_3_O_4_‐polystyrene microspheres with crosslinkers such as divinylbenzene and triallyl isocyanate [110]. These beads maintain the crystalline structure of the magnetic Fe_3_O_4_ core, while the amorphous polystyrene shell allows chemical modification.

**TABLE 4:** Representative examples of commercially available magnetic polystyrene beads (iron‐oxide embedded or coated). The information summarized in this table is derived from suppliers’ published product specifications. n.s., not specified.

MB surface	Size (µm)	Ms (emu/g)	Properties	Application	Ref.
Carboxyl, SA, Oligo(dT), Amine functional groups	11–3	n.s. (magnetite content 40%–60%)	Fast magnetic response, covalent protein/Ab coupling, high biotin‐binding capacity	Nucleic acid extraction, DNA/protein purification, mRNA isolation, cDNA library preparation, clinical IVD assays, immunoassays	[104]
PEG 300, PEG 3000, PEG 5000, PEG‐OMe	Nanoparticles (0.05–0.5) Microparticles (2–12)	n.s. for some products (WHM‐G141: *M*_s_ > 4.9; WHM‐G377: *M*_s_ > 110)	Little nonspecific binding, low toxicity, good biocompatibility, thermal, mechanical and chemical stability	Suitable for magnetic cell sorting, immunoassays, biosensors, magnetic resonance imaging, drug delivery	[105]
Unmodified or carboxyl	0.4, 0.8, 2, 3.5	SPM iron‐oxide crystals	Unmodified for hydrophobic ligand adsorption or covalent coupling by COOH groups	Magnetic cell separation, magnetic cell sorting, IVD immunoassays, affinity purification	[106]
Unmodified	10 (there are 0.1–5 variants)	n.s.	≥20% iron oxide, robust beads, suitable when adsorption‐based interactions are sufficient	Used for general separations, immunoassays, detoxification of biological fluids, hyperthermia and drug delivery	[107]
(CH2NH2)	5–50	n.s.	Loading capacity: 1.5–2.0 mmol/g	Adaptable for biomolecule immobilization, compatible with amide couplings	[108]

**FIGURE 10:**
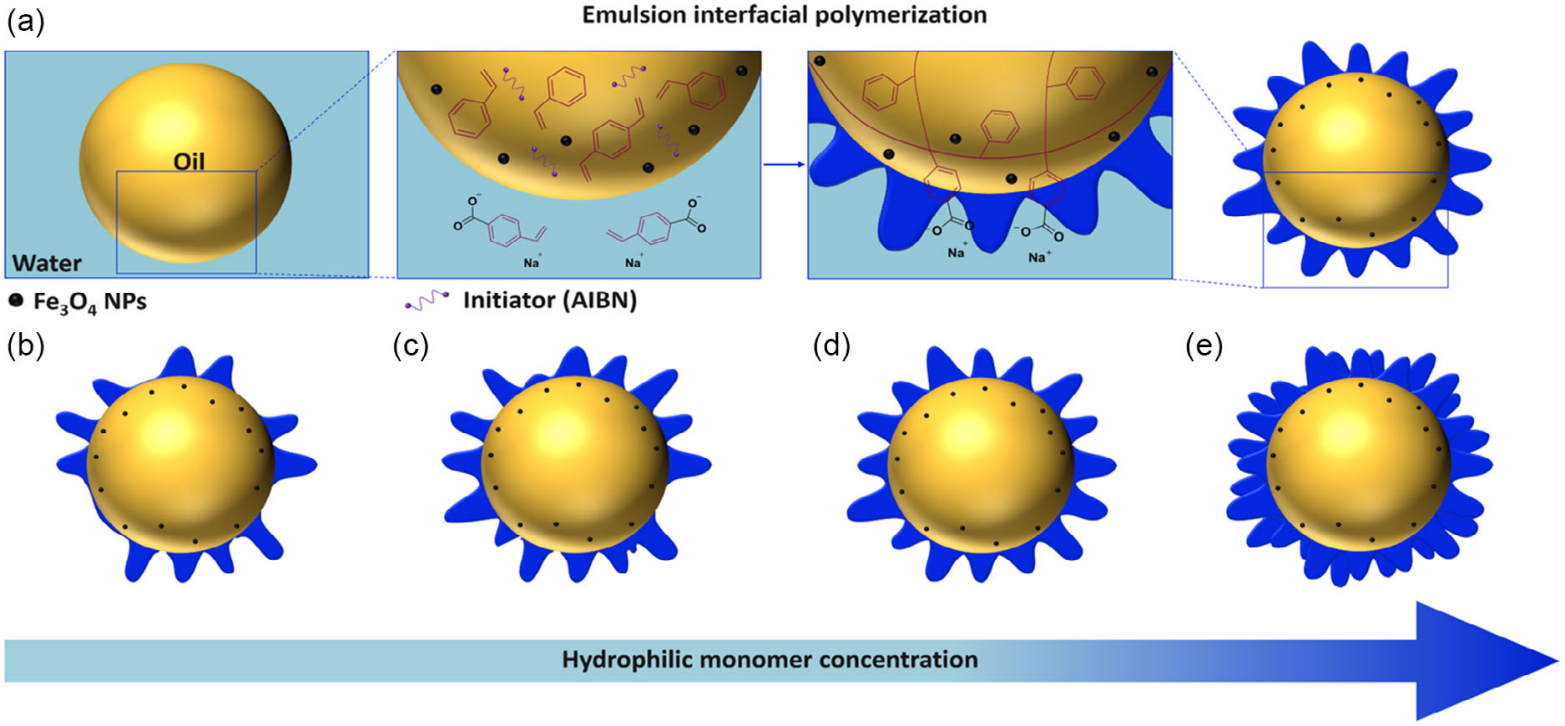
Illustration of the controllable synthesis of MBs via emulsion interfacial polymerization. (a) Schematic of the oil‐in‐water system and resulting MBs. (b–e) Regulation of nano‐filopodia length by varying the hydrophilic monomer concentration. Adapted with permission [103]. 2023 Wiley‐VCH GmbH.

Recently, Zhang et al. described a sophisticated strategy based on the interfacial polymerization regulated by electrostatic interactions to prepare microparticles with nanofractal structure. The approach involved a hydrophobic phase containing SPM oleic acid‐capped Fe_3_O_4_ NPs, hydrophobic monomers, cross‐linkers, and initiators, and the aqueous phase containing negatively charged hydrophilic monomers (Figure 10). Thermally initiated polymerization occurred at the interfacial, generating nanostructured polystyrene surfaces resembling cellular filopodia. The resulting MBs show tunable surface roughness and substantially enhanced cell‐capture efficiency compared with conventional smooth polystyrene beads [103].

Commercial polystyrene‐coated MNPs are commonly functionalized with carboxyl groups, enabling simple carbodiimide coupling. A representative example is the use of SPM NPs (203 nm) functionalized with carboxylic groups for EVs capture [111]. The synthetic strategy is based on the EDC/sulfo‐NHS activation of the carboxylic groups, followed by conjugation of Abs, such as anti‐CD9, and the quenching of residual active esters with BSA. This conventional workflow is widely used across polystyrene‐based liquid biopsy assays due to its reliability, efficiency, and compatibility with downstream magnetic separation. Beyond carboxyl groups, polystyrene MBs can be functionalized with sulfonic acids, amines, or other reactive moieties. As demonstrated by the sulfonated Fe_3_O_4_@SPS microspheres produced via chlorosulfonic‐acid treatment [110], the polymer shell serves as a highly adaptable platform for introducing defined chemical functionalities.

Taken together, polystyrene‐coated MBs showed high‐affinity surface chemistry, mechanical, and chemical robustness, controlled over morphology and topography, colloidal stability and efficient magnetic responsiveness. From high‐efficiency CTC capture, further enhanced by engineered nano‐topographies [103], to sensitive EV immunodetection [111], polystyrene provides a practical and adaptable foundation for rare BMs enrichment. Unlike silica, however, polystyrene shows no natural affinity for nucleic acids, meaning its value in liquid biopsy lies predominantly in selective capture via functional groups or affinity ligands, rather than direct DNA/RNA binding.

### 4.4 Streptavidin

Streptavidin (SA)‐coated MBs represent a robust and versatile class of affinity materials increasingly employed in liquid biopsy applications, particularly for the isolation of CTCs, EVs, and circulating nucleic acids. SA is a tetrameric protein known for its exceptionally high affinity toward biotin, one of the strongest noncovalent interactions known in nature, with an affinity of about 10^−14^ M^−1^. This interaction remains remarkably stable across a wide range of temperatures, pH values from 3 to 13, and diverse chemical environments, enabling efficient capture and enrichment of these rare and low‐abundance targets, and facilitating their detection and analysis [112]. These hybrid nanostructures typically consist of a magnetite core stabilized by a matrix, such as polymeric, Dex‐based, or silica coatings covalently functionalized with SA. Magnetite cores are generally synthesized by coprecipitation or thermal decomposition, followed by coating steps to prevent aggregation and to provide reactive functional groups. Covalent coupling, including carbodiimide‐mediated amide bonding (EDC/NHS), epoxy‐amine reactions, glutaraldehyde, thiol‐maleimide coupling, or silane‐mediated crosslinking on silica surfaces is used for the grafting of SA to MB surface. To reduce nonspecific adsorption of serum proteins and other biomolecules, blocking agents such as BSA, PEG or casein are used [113].

Despite their outstanding biorecognition properties, SA MBs can still exhibit limited nonspecific binding, particularly in samples containing other biotinylated molecules, which may require additional washing or purification steps. They are also relatively expensive for large‐scale applications, although their reusability after regeneration offsets part of the cost. Another limitation is the finite surface area, which constrains binding capacity when capturing large or highly abundant targets [114]. The design of MBs can be tuned across sizes, from submicron NPs optimized for nucleic acid capture to micrometer‐scale beads suitable for whole‐cell isolation, providing high surface area and compatibility with robotic liquid handling, flow cytometry, and microfluidic platforms [115].

Commercial SA MBs exhibit a range of physicochemical properties that influence their performance in liquid biopsy applications (Table 5).

**TABLE 5:** Representative types of commercially available SA‐coated magnetic particles. The information displayed in this table is collected from the suppliers’ websites. n.s., not specified.

MB surface	Size (µm)	Ms (emu/g)	Properties	Application	Ref.
SA quenched with BSA	2	SPM	Binding affinity 1000 pmol biotin/mg	Immunocapture and biopanning	[116]
Polysteren‐SA and blocked with BSA	2.7	SPM	Binding affinity >1200 pmol biotin/mg and >3000 pmol/mg for High Bind	Capture biotin‐labeled substrates, including antigens, Abs, and nucleic acids. Protein and nucleic acid pull‐down.	[117]
Polysteren‐SA	3–8	SPM	n.s.	DNA/RNA isolation, immunocapture, and potential CTC use with a proper ligand	[118]
SA on polymeric nanobead matrix with BSA protein	Nanobeads: 0.13; Microbeads: 1	n.s.	n.s.	Isolation of biotin‐labeled cells and EVs from blood, compatible with flow cytometry and CTC analysis	[119]
Dex‐SA and quenched with BSA	0.05–0.2	SPM	Binding affinity >20 ug biotin/mg	Isolation of biotinylated nucleic acids, Abs, or other biotinylated ligands and targets without columns or centrifugation.	[120]
Tosyl‐SA, blocked with BSA	1	n.s.	Binding affinity 950–1500 pmol biotin/mg MBs	Enrichment of targeted sequences in library preparations for NGS analysis. Suited for automated applications	[121]
Hydrophilic polymer‐SA	3	SPM	Binding affinity 400–600 pmol/mg MBs	Capture of biotinylated probes.	[122]
SA and blocked with BSA	0.05	n.s.	n.s.	Indirect cell labeling for CTCs; subcellular fraction separation	[123]
SA and 0.1% Recombinant BSA	1	SPM	Binding affinity 1000 pmol biotin/mg MBs	Capture biotin‐labeled substrates, including antigens, Abs, and nucleic acids. Protein and nucleic acid pull‐down	[124]
SA	0.05–0.2	SPM	Binding affinity 25–50 ug biotin/mg MBs	Cell separation, Immunoprecipitation, Immunoassay	[125]
Polysteren‐SA and blocked with BSA	1	PM	Binding affinity ≥1800 pmol biotin/mg MBs	Separation of biotin‐labeled molecules such as oligonucleotides, DNA fragments, proteins, peptides, glycoconjugates, and other antigens.	[126]
SA or polymer‐SA	0.2–9.9	n.s.	Binding affinity 700 pmol biotin/mg MBs	Immune assays and genomic assays for target detection.	[127]
SA	0.7–1.7	SPM	Binding affinity ≥3000 pmol biotin/mg MBs	Isolate biotin‐labeled DNA‐protein complex, capture single‐stranded biotinylated DNA oligos, and isolate biotinylated PCR products	[128]

Several SA MBs employ BSA as surface‐blocking agent, and their size ranges from tens of nanometers to several micrometers. MB size strongly influences both magnetic responsiveness and biotin‐binding capacity. High‐capacity MBs, such as those produced by Invitrogen and Agilent, are optimized for applications including target enrichment and nucleic acid capture, whereas while nanoscaled MBs from manufacturers such as BioLegend and CD Particles are particularly effective for isolation of biotinylated EVs or ligands in suspension assays. Differences in magnetite content, surface coatings (polymeric or hydrophilic), and surface chemistry further affect separation efficiency, biocompatibility, and adaptability to automated or high‐throughput workflows.

**FIGURE 11:**
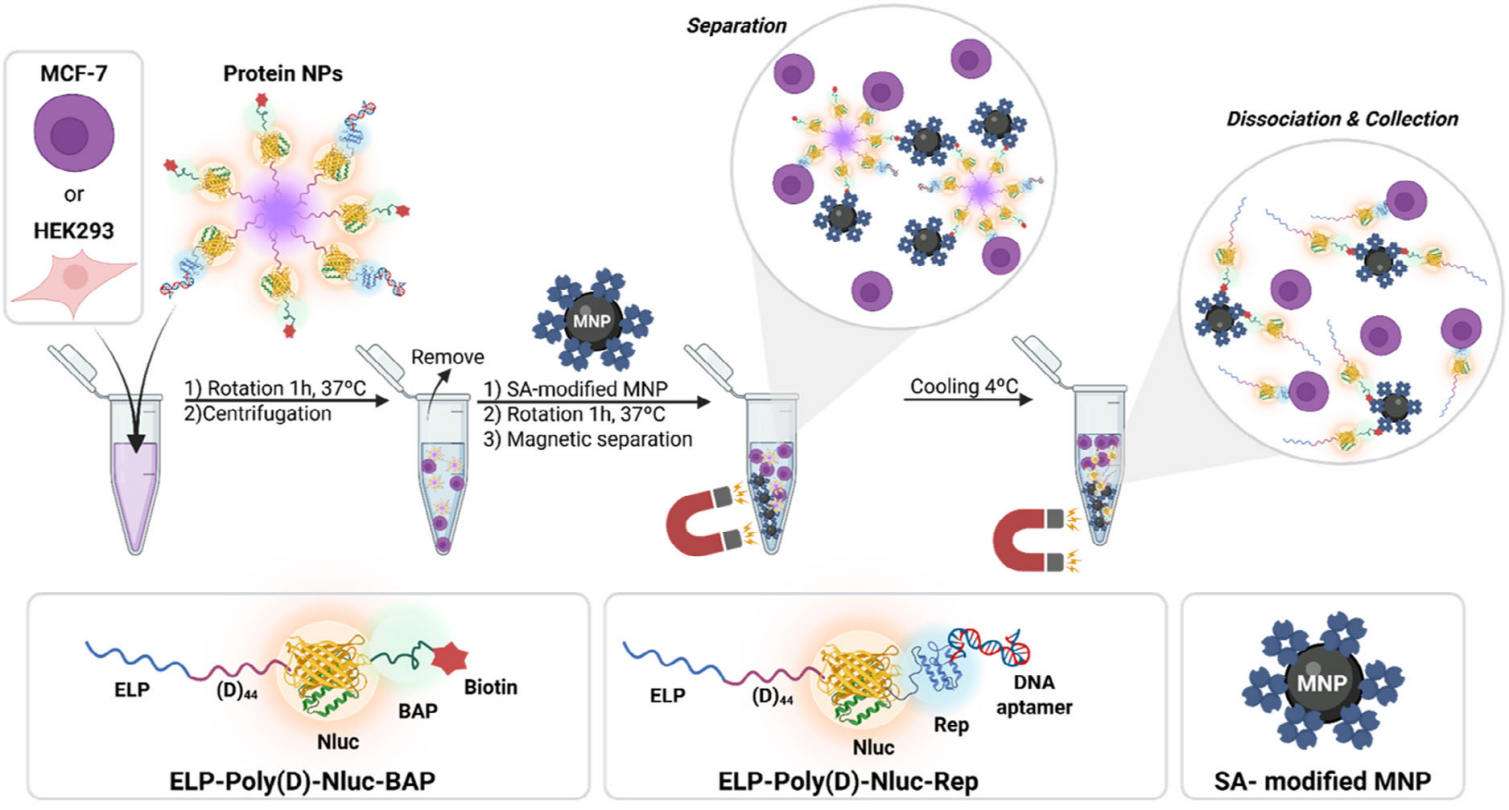
SA MBs employed for magnetic separation of target cells (MCF‐7 and HEK293) [130]. Created with Biorender.com.

Several studies illustrate the practical use of SA‐coated MBs in advanced diagnostic platforms. Chen et al. developed a NanoOctopus device composed of a magnetic microparticle functionalized with SA (diameter 4 μm), mimicking the octopus head and long single stranded DNA sequences anchored on the MB surface as tentacles for the capture of cancer cells from whole blood [129]. Nishida et al. employed temperature‐responsive protein MNPs to mediate the interaction between target cells and MNPs, enabling easy exchange of biological ligands, removal of MNPs by cooling, and effective cell isolation (Figure 11) [130].

In summary, SA MBs combine the strength and specificity of the biotin‐SA interaction with the controllable magnetic properties of iron oxide nanomaterials. Their modular design and adaptability across different size scales make them indispensable tools for the specific capture and enrichment of biotinylated targets in liquid biopsy workflows, facilitating the reliable isolation of ctNAs, EVs, and tumor cells with high sensitivity and reproducibility.

## 5 Magnetic Bead‐Based Capture of Biomarkers in Liquid Biopsy Applications

**FIGURE 12:**
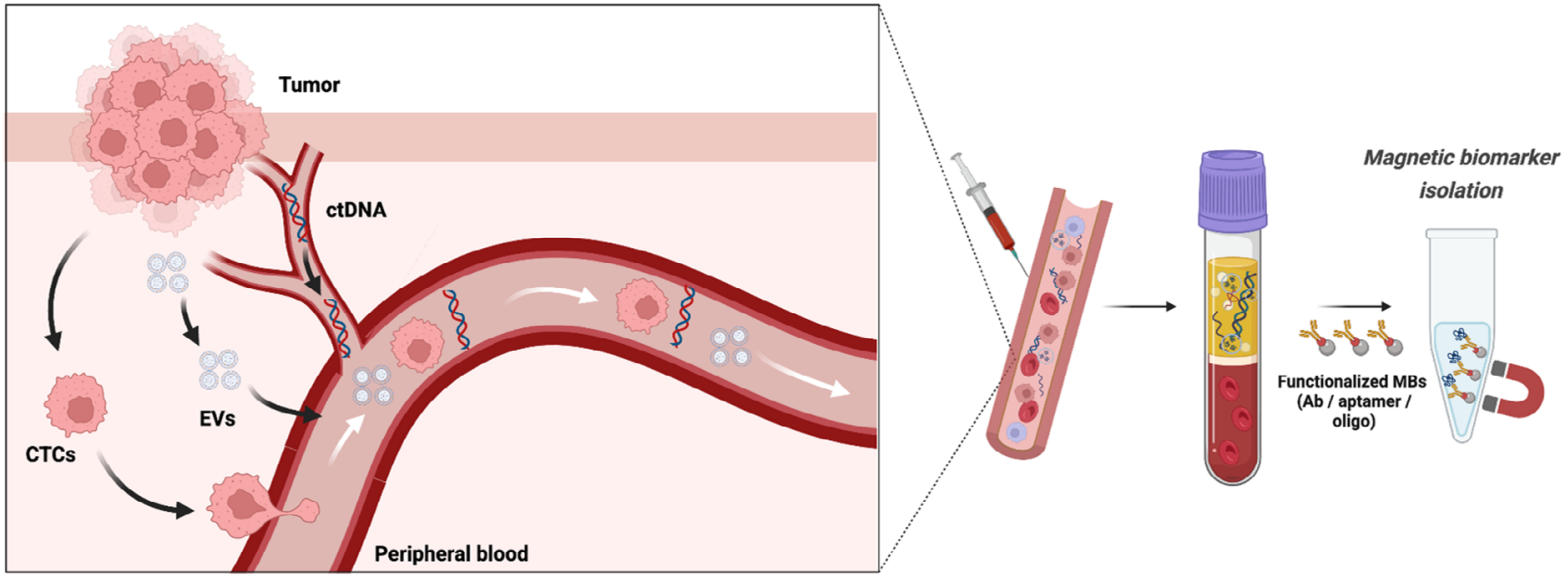
Tumor‐derived BMs in liquid biopsy and MBs based capture. Created with Biorender.com.

The efficient isolation and analysis of cancer BMs remain technically challenging because of their low abundance, heterogeneity, and the complexity of biological matrices. The most investigated BMs in cancer liquid biopsy include ctNAs, CTCs, and tumor‐derived EVs, which have been shed from tumor masses into the bloodstream, saliva, urine, CSF, among other peripheral fluids of patients (Figure 12). In the following section, an overview of the most representative literature papers on liquid biopsy MB‐based approaches will highlight the versatility of these tools, stemming from their tunable surface chemistry, compatibility with microscale and nanoscale formats, and ease of integration with microfluidic and analytical platforms.

### 5.1 Circulating Tumor DNA

ctDNA represents the tumor‐derived fraction of cfDNA and is a clinically relevant BM for cancer detection, treatment guidance, and disease monitoring [131]. ctDNA consists of short DNA fragments of ≈120 bp and has a short half‐life, ranging from 16 min to 2.5 h, which makes it difficult to distinguish from cfDNA released by normal cells. In the bloodstream, cfDNA derived from tumor cells typically accounts for less than 1% of the total cfDNA [132]. The concentration of cfDNA isolated from patient blood is typically low (10^3^−10^4^ copies/mL) in healthy or early‐stage patients, and higher in late‐stage patients. Considering the analytical challenges posed by ctDNA due to its low abundance (fM or lower) and short half‐life in complex biological fluids, the use of ultrasensitive biosensors is pivotal for its detection in blood. Chen et al. developed an electrochemical sensing device for the rapid detection of ctDNA from whole blood using gold‐coated MBs functionalized with a DNA probe that allows short‐ and long‐strand DNA hybridization. The biosensor exhibited a detection range from 2 aM to 20 nM for short strand ctDNA (22 nucleotides), with a detection limit of 3.3 aM and a range from 200 aM to 20 nM with a limit of detection of 3.3 aM in the case of long strand ctDNA [133].

Magnetic approaches for ctDNA analysis broadly encompass three interrelated strategies: i) MB‐based solid‐phase extraction of cfDNA from plasma; ii) sequence‐specific magnetic capture or enrichment of ctDNA using functionalized MBs; and iii) magnetophoresis‐enabled microfluidic systems that manipulate bead‐bound nucleic acids under controlled magnetic fields.

**FIGURE 13:**
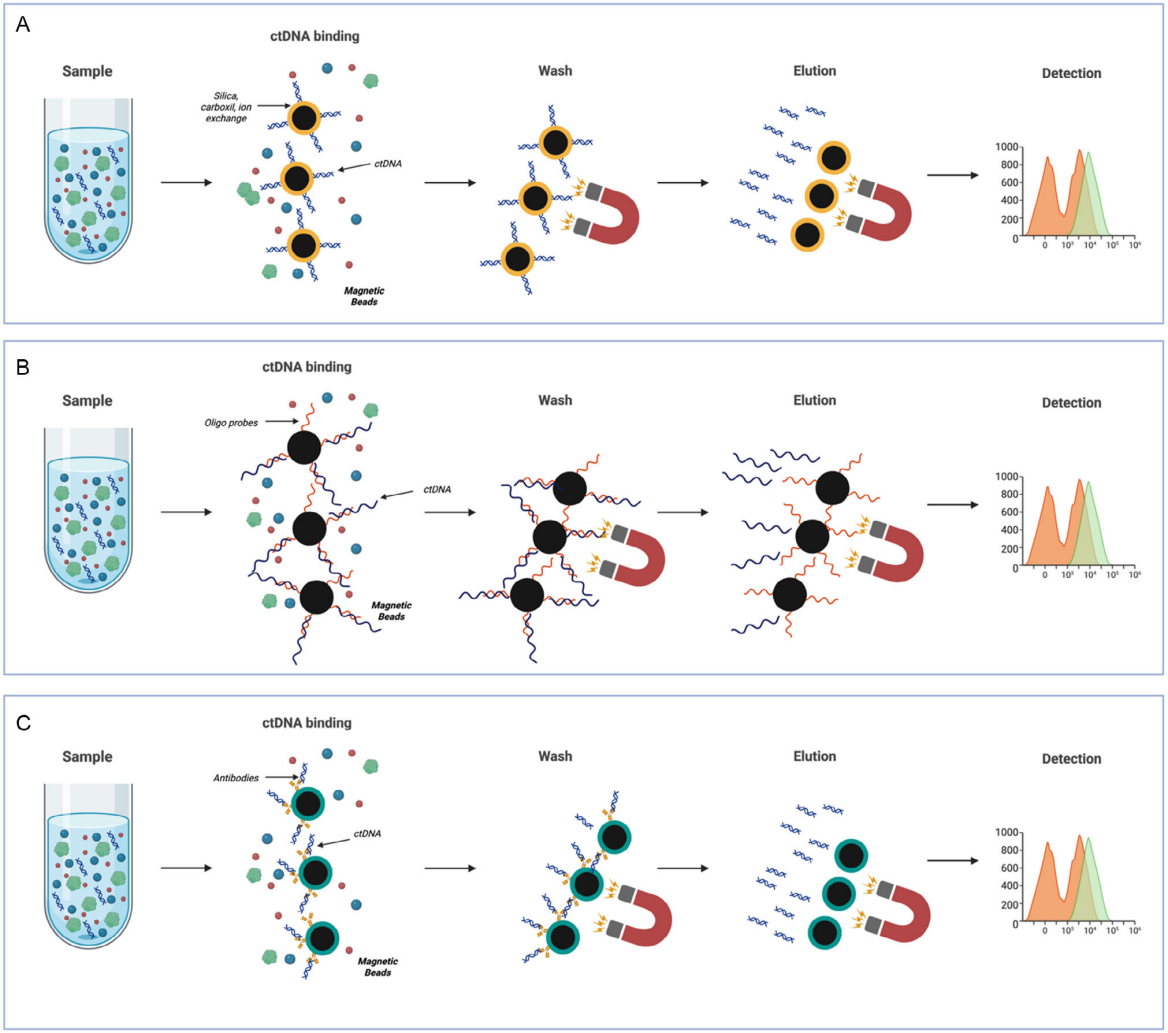
MBs‐based strategies for the capture and analysis of ctDNA. (A) Nonspecific binding of ctDNA using MBs functionalized with silica, carboxyl, or ion‐exchange surfaces, followed by magnetic separation, washing, elution, and downstream detection. (B) Sequence‐specific capture of ctDNA using oligonucleotide probe‐functionalized MBs, enabling selective hybridization, enrichment, and recovery of target DNA fragments. (C) Ab‐based approaches for ctDNA‐associated complex capture using immuno‐functionalized MBs. Created with Biorender.com.

In the first workflow, nucleic acids bind to functionalized MBs under defined buffer conditions, allowing subsequent magnetic separation and washing steps to remove impurities (Figure 13A). Two commercially available ctDNA extraction kits, MagMAX (ThermoFisher) and Maxwell RSC (Promega), employ this approach and are recommended by international guidelines [134].

In the second strategy, MNPs functionalized with oligonucleotide probes, peptide nucleic acids, or biotin–SA systems selectively hybridize to target DNA sequences. Following hybridization, magnetically captured ctDNA can be concentrated, washed, and either eluted or detected directly (Figure 13B). A notable example is the single‐particle collision electrochemistry (SPCE) biosensor developed by Shi et al. for rapid detection of ctDNA. In this system, ssDNA1 was grafted onto MB carboxylic groups via amide bonds and used as a probe for selective ctDNA capture. The CRISPR/Cas12a system cleaved ssDNA2, which interacted with Ag NPs through thiol groups. Cleavage by CRISPR/Cas12a released the Ag NPs, and their concentration was correlated with ctDNA content. The biosensor achieved a limit of detection of 4.2 fM with a linear range of 10 fM–1 nM [135]. Zhai et al. reported a flow cytometry‐based strategy using microsized SA MBs (New England Biolabs) for the capture of target DNA followed by fluorescence detection. This method combines enzyme‐free signal amplification with magnetic separation: target DNA initiates a hybridization chain reaction, forming fluorescent DNA structures that are subsequently captured by MBs and analyzed by flow cytometry, simplifying the capture and detection process. The authors reported a detection range of 5–100 nM with a detection limit of 2 pM [135].

The third strategy exploits the magnetic field gradients for the active transport of MBs bound to nucleic acids within a microfluidic environment. Many microfluidic platforms have been developed in which MNPs that are functionalized with Abs or proteins recognized by affinity target BMs such as cfDNA [136, 137, 138]. The separation of the BMs is obtained through magnetic manipulation, yielding high‐purity analytes that can be further used for downstream analysis (Figure 13C) [139, 140].

Compared to conventional ctDNA extraction methods, such as silica columns or precipitation‐based protocols, MBs‐based strategies offer improved scalability, automation compatibility, and flexibility in surface functionalization. MBs enable efficient enrichment of low abundance ctDNA from complex biological matrices and are readily integrated into microfluidic platforms and digital polymerase chain reaction (PCR) or sequencing workflows. While MNPs‐based and label‐free microfluidic approaches continue to emerge, MBs systems remain among the most mature and widely adopted technologies, with several commercial kits already available. Current trends focus on improving sensitivity for early‐stage disease, reducing nonspecific background cfDNA, and standardizing workflows for clinical implementation.

### 5.2 Circulating Tumor Cells

CTCs are intact malignant cells shed from primary or metastatic tumors into the bloodstream, providing direct insight into tumor heterogeneity and metastatic potential. Traditional CTC detection methods rely on epithelial markers such as epithelial cell adhesion molecule (EpCAM), which is commonly expressed in cancers including breast cancer, prostate cancer, pancreatic cancer, colorectal cancer, and hepatocellular carcinoma. However, certain tumor types, such as neurogenic tumors, do not present EpCAM or express it at very low levels [141, 142, 143]. Because of this limitation, additional BMs are required for CTC detection. Potential alternative BMs include human epidermal growth factor receptor‐2 (HER2), estrogen receptor, prostate‐specific membrane antigen, folate receptor and surviving [142]. In addition to BMs detection, CTC analysis can provide insights into cancer progression by revealing genome instability and specific mutations such as EGFR in non‐small cell lung cancer (NSCLC), APC and KRAS in colorectal cancer and BRCA1/BRCA2 or PIK3CA in breast cancer [144, 145, 146]. Copy number alterations in CTCs can also help track progression in lung, breast, and prostate cancers.

**FIGURE 14:**
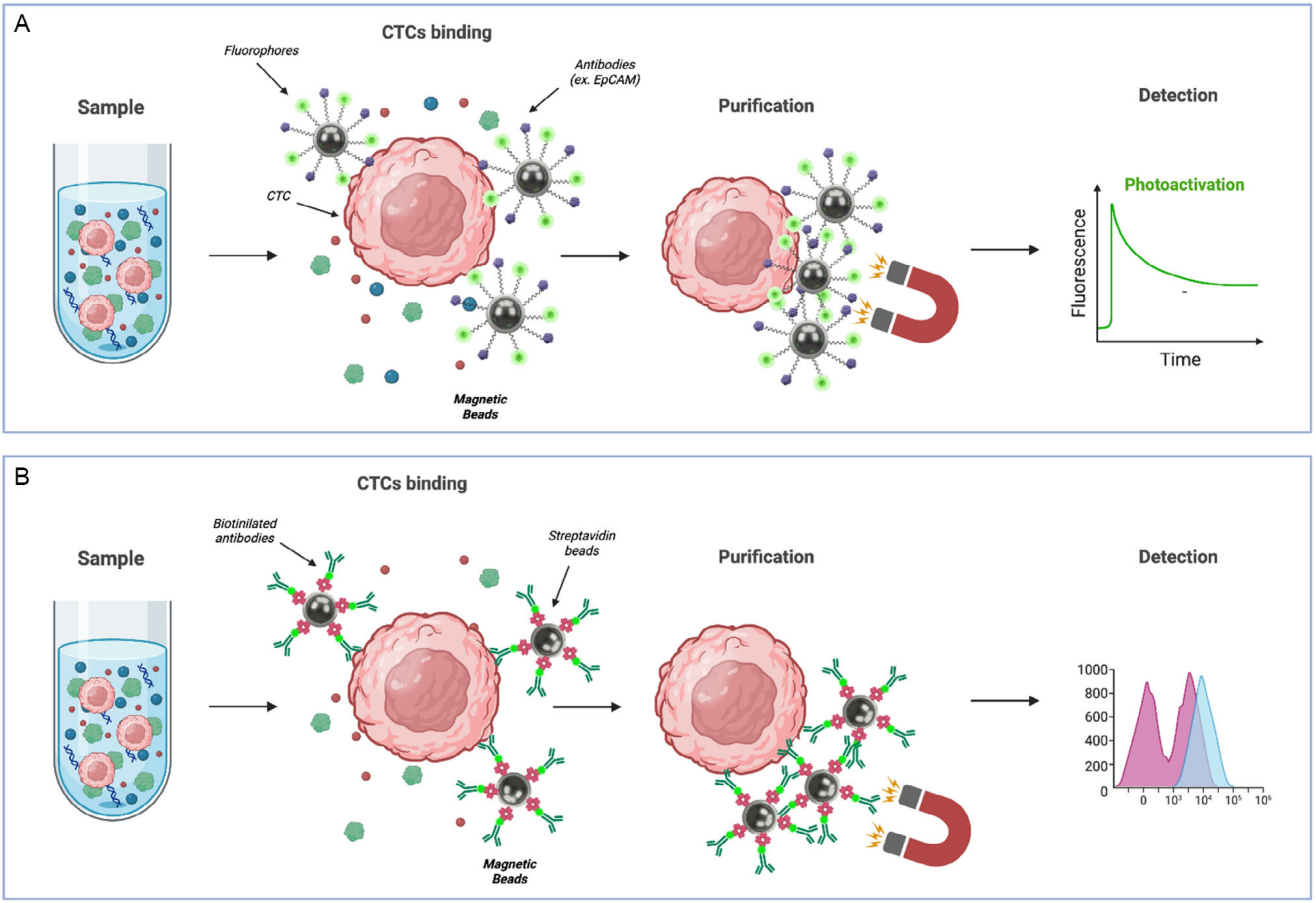
Immunoaffinity magnetic bead‐based capture of CTCs. Ab‐functionalized MBs, incorporating (A) fluorophore‐labeled Abs or (B) SA‐coated MBs with biotinylated Abs. Created with Biorender.com.

Techniques like fluorescence in situ hybridization can detect BMs such as ALK in NSCLC and HER2 in breast cancer, while DNA methylation analysis identifies patterns often associated with cancer [147, 148]. CTC isolation strategies expand into microfluidic and hybrid systems. Detection workflows typically begin with an enrichment step based on biological or physical properties, followed by immunocytological or molecular identification [149]. To improve the detection and enrichment of CTCs, a broad range of MB‐based strategies has been developed. As the clinical reference, the CellSearch system, based on EpCAM‐coated ferrofluid NPs, remains the only FDA‐approved assay for CTC enumeration in breast, prostate, and colorectal cancers [150]. Using Fe_3_O_4_ NPs modified with EpCAM Abs and fluorophores, CTC EpCAM‐mediated capture achieved an efficiency of 85% in liver cancer samples [141]. Magnetism, fluorescence, and biorecognition functionalities were integrated into single nano MBs (MB‐MLP‐EpCAM), with each function synergistically amplified (Figure 14A).

Building on these advances, microfluidic chips that integrate cell sorting, purification, and release regions have demonstrated high cell recovery (85%) and viability in blood samples for early clinical cancer diagnosis [151]. CTCs were captured by EpCAM modified MBs (EpCAM Apt‐MBs) and introduced into the designed microfluidic chip that integrated passive sorting and active sorting.

EpCAM‐dependent systems remain the most common, but their performance is limited in tumors with low EpCAM expression or undergoing epithelial‐mesenchymal transition, a process associated with cancer progression. This has motivated the development of multimarker, peptide‐functionalized, lectin‐based, and biomimetic magnetic platforms that expand capture capability beyond traditional epithelial markers. To overcome the limitations of single antigen targeting, several groups have developed multiantibody or multimodal magnetic platforms. Triple‐marker immunomagnetic beads (EpCAM, MUC1, EGFR) recovered ∼93% of SCLC cells from spiked samples and detected CTCs in 88.8% of patient samples [152]. Protein‐coated SA/tactin beads functionalized with anti‐EpCAM, anti‐HER2, and anti‐EGFR enabled multiplex capture and enumeration [153].

Given the antigen heterogeneity of CTCs and decreased EpCAM levels in cancers, EpCAM‐independent approaches including peptide‐ or lectin‐specific binding, have been also tested for CTC isolation [154]. Using this strategy, the carboxyl groups of MBs were exploited for the conjugation with Ulex europaeus agglutinin‐I (UEA‐I). UEA‐I lectin‐based MBs selectively captured α‐1,2‐fucosylated CTCs from colorectal cancer patients with 94% and 89% efficiency in medium and whole blood, respectively. In another study, hyaluronic acid (HA)‐modified MBs, specifically SiO_2_ MBs coated with polyacrylamide, captured HeLa cancer cells with 92% efficiency in microfluidic devices by exploiting CD44 overexpression [155].

Additional technologies include NanoVelcro platforms integrating antigen‐coated nanostructures and microfluidics to improve CTC isolation [156], MagSweeper, Cynvenio system using SA‐coated beads with biotinylated EpCAM Abs [157, 158], TiO_2_ nanoparticle immunoassays [159] for EpCAM‐positive CTCs, and bilayer microfluidics with polystyrene MNPs to retain targeted breast cancer cells [160].

Label‐free approaches such as microfiltration, density gradients, inertial focusing, and electrophoresis exploit physical differences to isolate CTCs and help overcome phenotypic heterogeneity [161]. A recent comparative analysis evaluated eight commercial SA MBs using an optimized Halbach‐based flow‐through immunomagnetic system designed to enhance capture of low EpCAM‐expressing CTCs. MBs in the 100–150 nm size range performed best, with MojoSort Streptavidin Nanobeads (BioLegend) achieving the highest capture efficiency for both high‐ and low‐EpCAM cell lines. The study also highlighted the importance of bead stability, identifying zeta potentials above 40 mV as ideal for colloidal stability (Figure 14B) [115].

MBs offer a balance between specificity and flexibility, enabling both EpCAM‐dependent and EpCAM‐independent capture through customizable surface functionalization. Compared to purely microfluidic or filtration‐based systems, MB approaches allow multiplex targeting and easier integration with downstream molecular and phenotypic analyses. Current trends emphasize multimarker capture, biomimetic coatings, and nanoscale MBs to address CTC heterogeneity and epithelial‐to‐mesenchymal transition in cancer.

### 5.3 Extracellular Vesicles

EVs are nanoscale (30–100 nm) lipid‐bilayer structures that mediate numerous cellular processes and have emerged as potential BMs for noninvasive disease diagnosis and monitoring treatment response, especially in cancer therapy [162, 163]. EVs are secreted by all cell types and can be detected in multiple biological fluids, facilitating minimally invasive sample collection. They carry specific proteins and nucleic acids, including miRNAs, derived from their parental cancer cells, which can reflect the organ of origin, organ‐specific metastasis, and overall disease state by studying their specific molecular signatures. Furthermore, their higher concentration and greater stability compared to other BMs have increased their interest as diagnostic and prognostic BMs in liquid biopsies for detecting various cancers, including lung cancer, pancreatic cancer, gastric cancer, prostate cancer, breast cancer, ovarian cancer, glioblastoma, and melanoma [164, 165].

EVs carry tumor‐specific molecules, including DNA and miRNAs, which can reveal cancer‐related mutations (e.g., KRAS, TP53) and serve for early detection. Oncogenic miRNAs, such as miR‐21, miR‐155, miR‐17‐92, and miR‐1246 promote cancer progression, while reduced levels of tumor‐suppressor miRNAs (e.g., miR‐146a, miR‐34a) correlate with poor prognosis [166].

**FIGURE 15:**
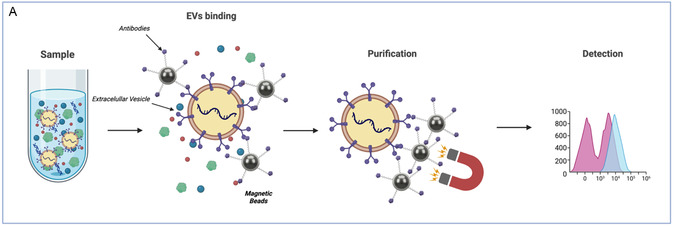
MBs isolation of EVs via surface immunochemistry. Created with Biorender.com.

Traditional EV isolation techniques, such as ultracentrifugation and polymer‐based precipitation, are limited by low purity, long processing times, and poor reproducibility. In contrast, MB‐based approaches provide higher specificity through immunoaffinity capture and enable rapid isolation compatible with small sample volumes (Figure 15). Compared to size‐based or label‐free methods, MB‐based platforms facilitate selective enrichment of disease relevant EVs subpopulations and seamless integration with biosensing and microfluidic systems. Emerging trends include the use of nanoscale MBs, aptamer‐based capture, and multiplexed detection strategies to improve sensitivity and clinical applicability [167, 168].

Recent reviews have comprehensively analyzed advances in EV isolation and release using functionalized MBs [26], identifying the main limitations that hinder efficient EV separation using MBs. Key challenges include: a) insufficient magnetic separation, which prevents the complete recovery of EV‐MB complexes from the sample; b) the intrinsic complexity of clinical fluids, reducing the recognition efficiency of Abs, aptamers, and ligands; c) aggregation of MBs; d) the pronounced heterogeneity of EV populations; e) inefficient EV release or potential EV damage during the release process; f) low EV purity; g) the high cost of some recognition elements and the limited recyclability of functionalized MBs; and h) the absence of standardized EV isolation protocols. Overall, although EVs are stable, abundant in body fluids, and carry diverse molecular information reflecting tumor microenvironment dynamics and mechanisms of drug resistance, the isolation of cancer‐specific populations remains technically challenging.

## 6 Conclusions and Future Perspectives

MB‐based technologies represent a central tool in liquid biopsy due to their versatility, scalability, and compatibility with a wide range of BMs, including ctDNA, CTCs, and EVs. MBs enable selective capture and enrichment of low‐abundance BMs from complex biological fluids, significantly improving analytical sensitivity and facilitating downstream molecular analyses. Their integration with microfluidic platforms, sequencing technologies, and biosensing systems has further enhanced their applicability in precision oncology. The main advantages of MBs in liquid biopsy applications include high capture efficiency, ease of functionalization with Abs, aptamers, peptides, or lectins, and compatibility with automation and clinical workflows. MBs support both microscale and nanoscale strategies, allowing flexible assay design depending on the target BMs and clinical need. These properties make MBs particularly attractive for longitudinal monitoring, minimal residual disease detection, and personalized treatment guidance. Despite these advances, several challenges remain, including BMs heterogeneity, nonspecific binding, limited sensitivity in early‐stage disease, and the lack of standardized protocols across platforms. Additionally, while nanoscale MBs offer improved surface‐to‐volume ratios and enhanced interaction with small BMs, their stability, reproducibility, and clinical translation still require further optimization. Addressing these challenges will be essential for the broader clinical implementation of MBs‐based liquid biopsy technologies. MBs have demonstrated significant potential in enhancing liquid biopsy performance through selective enrichment, multiplexing capability, and integration with advanced analytical platforms. While ctDNA analysis is already approaching routine clinical implementation, MB‐based technologies for CTC and EVs isolation continue to evolve rapidly. Future progress will depend on improving assay standardization, sensitivity in low‐BM settings, and clinical validation across cancer types. Continued innovation in MBs design, surface chemistry, and microfluidic integration is expected to further expand their impact on precision oncology.

## Funding

HORIZON‐MSCA‐2021‐DN‐01 (101072462).

## Conflicts of Interest

The authors declare no conflicts of interest.
